# Degradation of Polymer-Drug Conjugate Nanoparticles Based on Lactic and Itaconic Acid

**DOI:** 10.3390/ijms232214461

**Published:** 2022-11-21

**Authors:** Mai Dang Le Vuong, Mohamed Haouas, Merve Seray Ural, Didier Desmaële, Charlotte Martineau-Corcos, Ruxandra Gref

**Affiliations:** 1 CNRS, Institut Lavoisier de Versailles, UVSQ, Université Paris-Saclay, 78000 Versailles, France; 2 CNRS, Institut des Sciences Moléculaires d’Orsay, Université Paris-Saclay, 91405 Orsay, France; 3 CNRS, Institut Galien Paris-Saclay, Université Paris-Saclay, 91400 Orsay, France

**Keywords:** drug delivery, polymer nanoparticles, poly(lactic acid), itaconic acid, NMR spectroscopy

## Abstract

Tuberculosis (TB) is still a significant threat to human health. A promising solution is engineering nanoparticulate drug carriers to deliver anti-TB molecules. Itaconic acid (ITA) potentially has anti-TB activity; however, its incorporation in nanoparticles (NP) is challenging. Here we show an approach for preparing polymer-ITA conjugate NPs and a methodology for investigating the NP degradation and ITA release mechanism. The conjugate was synthesized by the two-directional growing of polylactic acid (PLA) chains, followed by capping their extremities with ITA. The poly(lactate)-itaconate PLA-ITA was then used to formulate NPs. The degradation and drug release processes of the polymer conjugate NPs were studied qualitatively and quantitatively. The molecular structures of released species were characterized by using liquid NMR spectroscopy and mass spectrometry. We discovered a complex NP hydrolysis process forming diverse oligomers, as well as monomeric lactic acid (LA) and drug ITA. The slow degradation process led to a low release of free drugs, although raising the pH from 5.3 to 7.4 induced a slight increase in the amounts of released products. TEM images showed that bulk erosion is likely to play the primary role in the degradation of PLA-ITA NPs. The overall results and methodology can be of interest for understanding the mechanisms of NP degradation and drug release of this new polymer-drug conjugate system.

## 1. Introduction

Tuberculosis (TB) is one of the world’s top ten causes of death and is the deadliest infectious disease, according to the 2021 global report of WHO on TB [1]. It is caused by *Mycobacterium tuberculosis* (*Mtb*), a bacteria that infects about one-quarter of the world’s population, leading to 1.5 million deaths yearly [1]. *Mtb* primarily affects the lungs by spreading through the air in the form of aerosol droplets emitted by an infected person [2]. Sometimes, *Mtb* can persist in pulmonary alveolar macrophages in a dormant state for a long period, so the risk of reactivation exists even decades after infection.

Current first-line treatment for drug-susceptible TB consists of a 2-month initiation phase with the antibiotics isoniazid (INH), rifampicin (RIF), and pyrazinamide (PZA) [3,4,5,6]. This drug regimen can successfully treat approximately 85% of TB-infected patients but is associated with numerous side effects frequently causing poor patient compliance. In addition, irregular drug supply and a lack of supervision contribute to the emergence of drug-resistant *Mtb* strains [7].

There is a clear need to develop more efficient treatments to eradicate TB. Increasing focus is put on nanoparticulate drug carriers targeting *Mtb* in alveolar macrophages, as nanoparticles (NPs) have many advantages, such as drug (co)encapsulation and protection bypassing biological barriers to reach the biological targets [8,9,10,11,12]. Therefore, NPs can allow the administration of novel and potent anti-TB drug molecules.

In this context, the original active ingredient in this study is itaconic acid (ITA), a human metabolite playing an important role in the immune response [13] and with potential antimicrobial and anti-inflammatory activity [14]. It has never been used yet for TB treatment, but Michelucci et al. demonstrated that ITA might inhibit the growth of *Mtb* [15]. Nevertheless, ITA, as a free drug, cannot penetrate inside the infected cells. That is why it is necessary to design a suitable ITA nanocarrier.

However, ITA incorporation in NPs is a challenge, given the high solubility of this molecule in aqueous solutions and its tendency to crystallize. For dealing with these issues, a significant research trend is the synthesis of drug bioconjugates that self-assemble into NPs, leading to excellent incorporation efficiencies. For example, various drugs were conjugated with squalene, resulting in amphiphilic derivatives that spontaneously formed NPs [16]. Similarly, drugs were conjugated with polymers. Tong and Cheng carried out pioneering studies in the “drug-initiated” synthesis of polymers [17]. This method has been proposed to prepare polymer prodrugs using either native functional groups of the drug for ring-opening polymerization or pre-functionalized drugs for reversible deactivation radical polymerization techniques [17,18,19,20,21]. Pipemidic acid (PIP), an antibacterial drug, was successfully conjugated to poly(ε-caprolactone) (PCL) which self-assembled to produce NPs [22]. The PIP-PCL conjugate was synthesized employing the “drug-initiated” method in which the drugs were initiators for the polymerization.

Additionally, biodegradable polymers such as poly(lactic acid) or polylactide (PLA), poly(D,L-lactic-co-glycolic acid) (PLGA), and polyacrylic acid (PAA) are some of the most common biomaterials to make drug-loaded NPs [23,24,25,26]. In particular, PLA, whose monomer is lactic acid (LA), is a popular drug carrier because of its biocompatibility and biodegradability [24,27]. It was chosen to synthesize the pH-responsive polymer-drug conjugate NPs made of poly(lactic acid)-itaconate PLA-ITA. As the environment inside the TB-infected cells or granuloma has a more acidic pH than that inside normal cells [28,29], successful NP degradation and ITA release upon changing environmental pH will result in targeted intracellular delivery of ITA.

This study aims to design new polymer-drug conjugate NPs and to study their degradation mechanism. A polymer synthesis step is followed by a formulation optimization procedure and analysis of possible degradation products. 

Nuclear magnetic resonance (NMR) spectroscopy has attracted attention for the characterization of micelles and colloids for pharmaceutical applications [30,31,32]. As a classical structural tool, it has become a promising technique for studying drug delivery processes of nanocarriers [33,34,35]. For instance, ^1^H and ^13^C solution and solid-state NMR studies have shown that amphiphilic poly(lactic acid)-poly(ethylene glycol) PEG-PLA copolymer forms solvent-dependent structures [36]. Herein, we investigate the degradation processes of PLA-ITA conjugate NPs mainly using liquid ^1^H and ^13^C NMR spectroscopy to identify and quantify the released products. The methodology proposed here could be helpful for other materials made by the “drug-initiated” method.

## 2. Results and Discussion

### 2.1. Synthesis of PLA-ITA Polymer-Drug Conjugate

**Figure 1:**
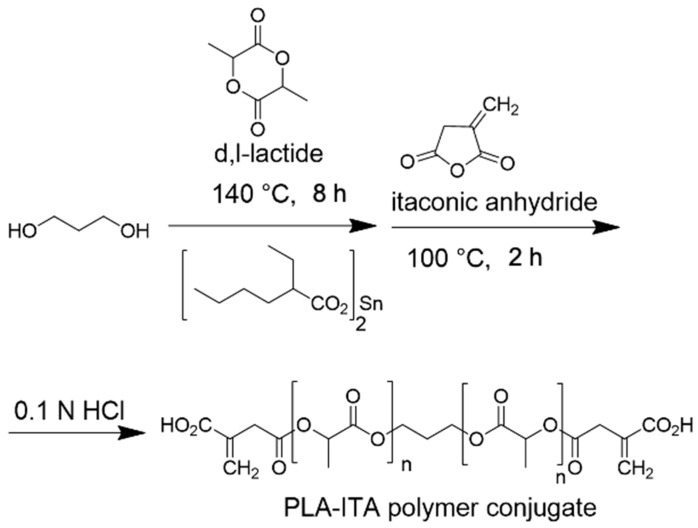
Synthetic representation for the synthesis of two-directional poly(lactic acid)-itaconate, denoted as PLA-ITA.

The synthesis of ITA-terminated poly(L-lactic acid) oligomers was first reported by Okuda et al. in 2012 [37]. They showed that it was possible to ring open itaconic anhydride by the OH terminal group of a PLA chain by a one-pot process using a tin octanoate catalyst. A reasonable chemoselectivity (87:13) for the more reactive C-5 carbonyl was observed, and β-mono ester of itaconic acid was produced. To maximize the amount of incorporated ITA while maintaining the self-assembly capacity of the oligomer, we planned a two-directional growth of the polymeric chain to get two terminal hydroxyl groups available for reaction with itaconic anhydride. Thus, conventional tin-catalyzed PLA synthesis using D,L-lactide ring-opening polymerization was performed starting from 1,3-propanediol as the initiator (Figure 1). After completion, the mixture was cooled, and an excess of itaconic anhydride was added. After 3 additional hours of heating at 100 °C, the crude material was acid-washed and purified by precipitation affording the desired material as a white solid. The material was characterized by ^1^H and ^13^C NMR spectroscopy, IR spectroscopy, and mass spectrometry to determine its structure (see Section 1 in Supporting Information and Figures S2–S4).

The ^1^H NMR spectrum of the obtained product (Figure S1) shows the characteristic peaks of the repeating unit of lactic acid at 5.17 and 1.57 ppm and the signals of the propane diol at 4.20 and 1.99 ppm. The chemical shift of lactate in PLA-ITA is similar to that in PLA polymer alone and agrees with the literature [38]. The signature peaks of the two terminal itaconate units displayed at 5.88 and 6.45 ppm correspond to the protons of the methylidene groups =CH_2_. The smaller singlet at 5.83 ppm can be tentatively assigned to the minor α-mono ester of itaconic, as observed previously [37]. Peak integration indicated an average molecular weight of Mn = 3850–4050 g.mol^−1^, roughly corresponding to 25 lactic units for both hydroxyl groups of the propanediol.

In short, PLA-ITA polymer-drug conjugate was synthesized successfully.

### 2.2. Preparation of PLA-ITA Polymer-Drug Conjugate Nanoparticles

Once the polymer PLA-ITA was produced, we optimized the formulation of PLA-ITA NPs, based on the NPs’ mean hydrodynamic diameters and polydispersity (Table S1). Among the tested formulations, single emulsion using dichloromethane (DCM) as an organic solvent and PVA as an emulsifier, gave best results while avoiding polymer precipitation. The obtained PLA-ITA NPs had an average diameter of 331 ± 12 nm and a polydispersity index (PDI) of 0.09 ± 0.05.

Nevertheless, the single emulsion formulation was not suited for the degradation study. In a first attempt, PLA-ITA NPs prepared by single emulsion were incubated at pH 7.4 and 5.3 for over one month. The obtained supernatant was measured by liquid NMR spectroscopy. The ^1^H NMR spectra (Figure S5) show difficulties in accurate analysis. Firstly, the peaks of interest of ITA in the region 5.5–6.5 ppm have very low intensity, hindering both qualitative and quantitative characterization. This low intensity is due to low ITA loading in 7.6 wt% of PLA-ITA, which corresponds to maximum ITA release below 3 mmol L^−1^. However, it is not easy to increase the wt% of ITA because it will increase the solubility of the bioconjugates in aqueous media, risking its ability to form NPs in water. The second issue is that the signals of interest of LA in the regions 1.1–1.8 ppm and 4.0–5.2 ppm strongly overlap with the resonances of PVA at 1.7 and 3.9 ppm. The signals of PVA are very intense compared to those of ITA and LA due to the high amounts (4 mg/mL) of PVA in the NPs suspension [39].

To avoid these issues, PLA-ITA NPs were prepared by nanoprecipitation in small volumes without using a PVA emulsifier. The attained mean hydrodynamic diameter was 343 ± 36 nm, and the PDI was 0.49 ± 0.07. In addition, the concentration of the analyzed sample was increased to the maximum to improve the NMR sensitivity. Specifically, samples of degradation products were freeze-dried, and the obtained precipitates were redissolved again in a minimal amount of D_2_O.

### 2.3. Identification of Degradation Products of PLA-ITA Nanoparticles

**Figure 2:**
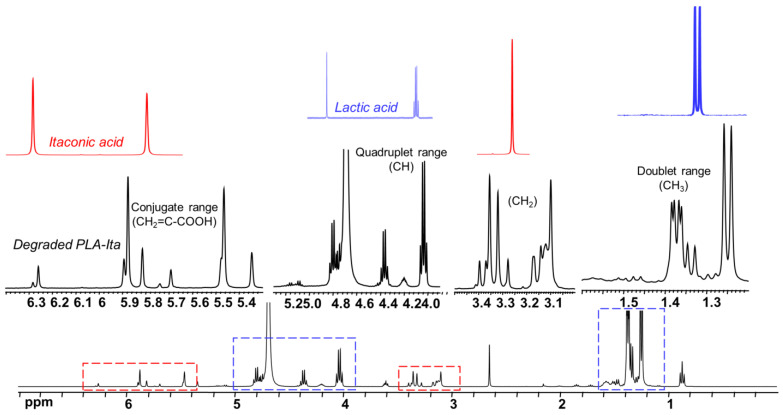
^1^H NMR spectrum of released product from PLA-ITA incubated for 2 months in PBS at pH 7.4 compared to spectra of aqueous solutions of ITA (red lines) and LA (blue lines).

The ^1^H NMR spectrum of freeze-dried degradation supernatant of PLA-ITA nanoprecipitation in phosphate buffer saline (PBS) at pH 7.4 after two months was displayed in Figure 2. It presents several peaks assigned to methylidene protons =CH_2_ of ITA in the region 5.7–6.6 ppm, contrasting with two singlets in the same region of free itaconic acid. There are also more signals assigned to the methylene -CH_2_- protons in the region 3.25–3.70 ppm, in comparison with one peak in the spectrum of itaconic acid. Such an increase in NMR signature peaks was not observed when incubating ITA in similar conditions of PLA-ITA degradation (Figure S6), indicating that this phenomenon is due to the degradation reaction of PLA-ITA and not to the interaction between ITA and phosphate salts. Likewise, there are several quadruplets and doublets corresponding to LA’s methine protons and methyl protons in the region 4.0–5.5 and 1.2–1.8 ppm, respectively. Such multiplication of LA’s NMR signatures was also detected in lactic acid aqueous solution incubated at similar conditions of PLA-ITA degradation (Figure S6). It was evidenced by Espartero et al. that LA in an aqueous solution can exist in the form of different oligomers, e.g., monomer, dimer, and trimer, resulting in multiple characteristic peaks in ^1^H NMR [40]. In agreement with these findings, the analysis of our degradation mixtures by ^1^H COSY NMR revealed a mixture of at least three lactate species in monomeric, dimeric, and trimeric forms.

**Figure 3:**
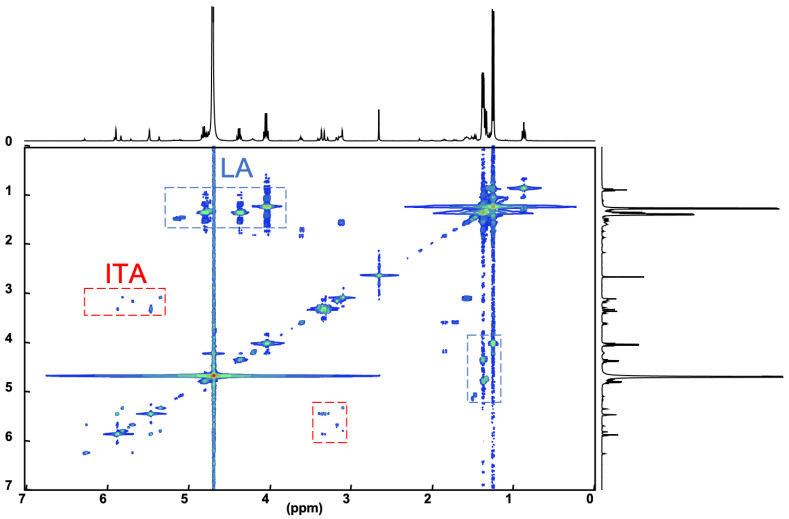
^1^H-^1^H COSY NMR spectrum of water-soluble degradation products of PLA-ITA nanoprecipitation incubated in PBS at pH 7.4 for 2 months.

**Figure 4:**
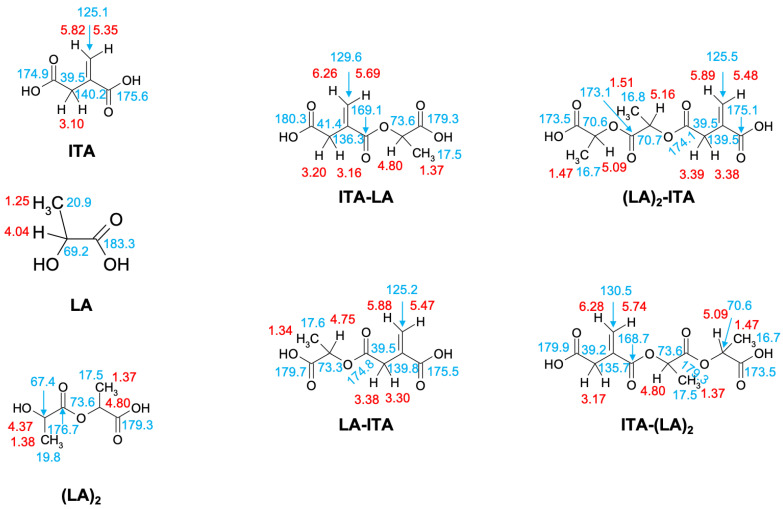
The main water-soluble degradation products of PLA-ITA NPs in PBS at pH 7.4, detected by NMR spectroscopy (^1^H in red and ^13^C in blue).

Therefore, we hypothesized that the degradation reaction of PLA-ITA also produces such oligomers between ITA and LA. To confirm this hypothesis and explicitly assign these different peaks of ITA and LA, 2D NMR correlation measurements were carried out. ^1^H-^1^H COSY NMR measurement (Figure 3) helped to distinguish different peaks in 1D ^1^H by showing the correlation pairs between methylidene =CH_2_ and methylene -CH_2_- protons of ITA fragments (Figure S7) and between methine -CH and methyl -CH_3_ protons of LA fragments (Figure S8). Additionally, ^13^C-^1^H HSQC and HMBC NMR (Figures S9–S14) gave complementary information for the NMR assignment through the correlation pairs between different types of protons with carboxylate -COO, double-bonded C=CH_2_, and methylene -CH_2_. We identified 5 groups of correlated signals (4 proton signals and 5 C signals) for ITA fragments and 6 groups of correlated signals (a couple of proton signals and 3 C signals) for LA fragments. These data and possible structures of PLA-ITA degradation products are gathered in Figure 4.

Likewise, the existence of oligomers made of itaconate and lactate was also demonstrated by MS spectrometry. The mass spectra of degradation products in PBS at pH 7.4 for two months (Figure S15) showed peaks of (−1) negative fragments of lactic acid (*m*/*z* 89.3) and itaconic acid (*m*/*z* 129.1), as well as peaks of mixed esters between ITA and LA monomers or propane-1,3-diol (Table 1).

The remaining parts of the PLA-ITA NPs were analyzed by MALDI MS. Some main peaks in the positive mode MALDI spectra of original and degraded PLA-ITA were listed in Table 2. In the spectrum of degraded PLA-ITA (Figure S16) recorded without added cations, the peaks for [M+Na]^+^ and [M+K]^+^ were present because the instrument’s glass chamber is made of Na and K. When Na^+^ was added, the peaks for [M+K]^+^ decreased their intensity, thus supporting the peak assignments. Moreover, the spectrum for the pH 5.3 sample shown in Figure S16 exhibits signals of PLA-ITA with two-time shorter chain lengths than the original one (Table 2). This result indicates that: (i) the PLA-ITA NPs are degraded into smaller fragments, consistent with the NMR and MS data, but (ii) the degradation is still incomplete after 1–2 months.

From the evidence of NMR and MS, it appears that the degradation of PLA-ITA NPs not only produced itaconic acid and lactic acid but also created oligomers of ITA-LA and ITA-propane-1,3-diol. The mechanism of base-catalyzed ester hydrolysis seems to be random cleavage of the ester bonds and polymer chains. As a result, oligomers of different chain lengths were formed.

Furthermore, quantitative ^1^H NMR allowed us to determine the % release of ITA drug and lactic acid and studied the effect of pH on drug release and NPs degradation. Table 3 shows that ITA-containing and LA-containing species formed faster at neutral pH 7.4 than at acidic pH 5.3. It took 2 months for the degradation at pH 5.3 to produce a similar level of % ITA release to that of degradation at pH 7.4 after 1 month. Conventionally, ester hydrolysis is usually catalyzed by acidic H^+^ cations [41]. However, knowing that the starting pH of original PLA-ITA NPs is slightly acidic (pH = 5–6), incubation of PLA-ITA at pH 7.4 caused a bigger pH change than at pH 5.3. In this case, the hydrolysis of the ester bonds between LA and ITA probably followed a B_AC_2-type mechanism catalyzed by OH^-^ anion [42]. The slow and prolonged release of ITA conjugated with PLA NPs can be more advantageous than the burst release of unconjugated drugs [43].

### 2.4. Degradation Mechanism of PLA-ITA Nanoparticles

**Figure 5:**
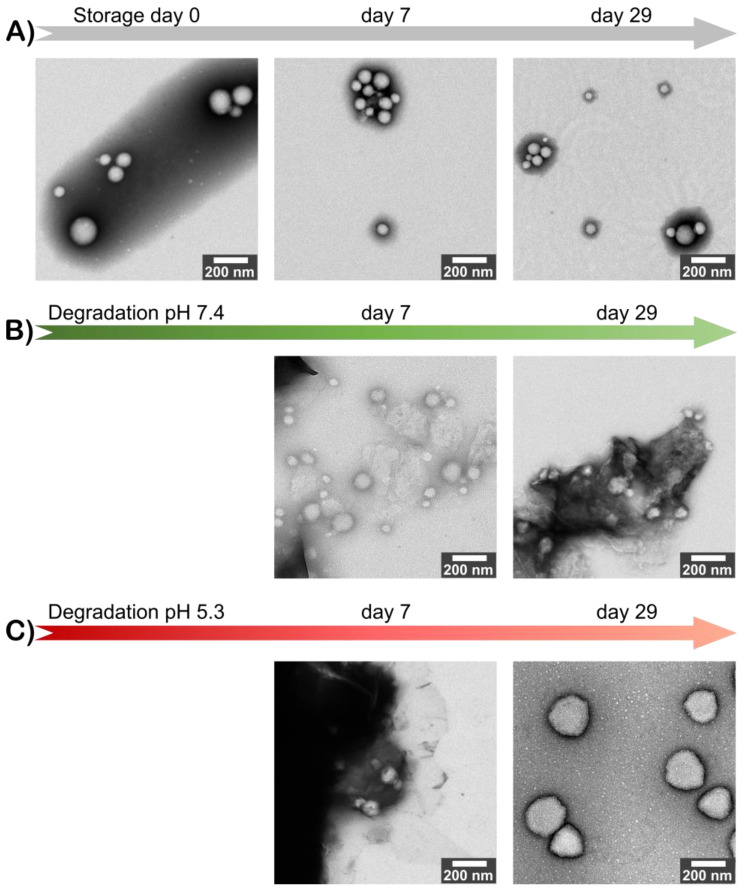
TEM images of PLA-ITA NPs in a 1-month course of (**A**) original and degraded at (**B**) pH 7.4 and (**C**) pH 5.3.

To get a global view of how the degradation process happens, we monitored the size, morphology, and stability of original and degraded PLA-ITA NPs for 2 months. On the one hand, the size distribution and zeta potential of the original PLA-ITA NPs stored at 4 °C for 2 months had less than a 10% difference. On the other hand, the degraded PLA-ITA NPs suspension aggregated, whose size became too big to be measured by DLS. The ZP magnitude of degraded PLA-ITA NPs decreased 2-times more than that of the original NPs (Figure S17). As we could not determine the size of degraded NPs by DLS, we used TEM to observe the evolution of NPs size and morphology. Figure 5 shows that as time progressed, while the original NPs kept their morphology, the PLA-ITA NPs degraded at pH 7.4 and became transparent. This NP transformation could be a sign of a bulk erosion mechanism characterized by a fast water diffusion into the NPs followed by a slower water hydrolysis reaction [44]. Thus, NPs could undergo bulk degradation, maintaining their size roughly unchanged. However, degradation at pH 5.3 seemed to produce swollen NPs (Figure 5C). Note that this swelling phenomenon is not observed in a control experiment with pure PLA NPs without the presence of ITA (see Figure S18), suggesting that the swelling was related to the presence of ITA within the NPs. 

**Figure 6:**
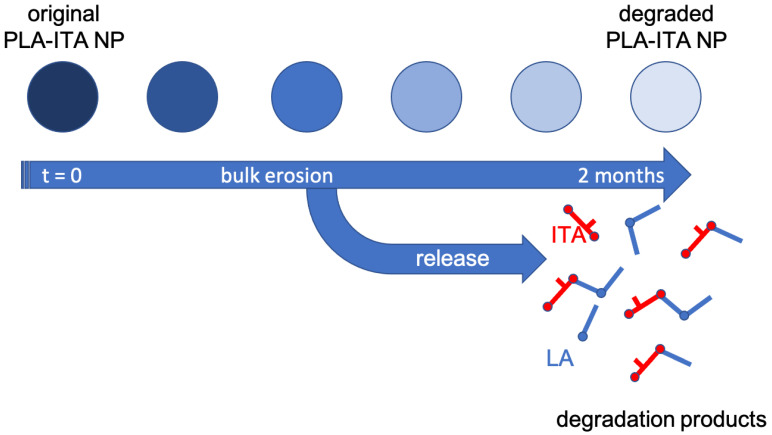
Schematic representation of degradation and drug release at pH 7.4 of PLA-ITA polymer-conjugate NPs following bulk erosion mechanism with limited species release.

Figure 6 proposes the scheme of the degradation mechanism of PLA-ITA polymer conjugate NPs at physiological pH, as revealed at the macroscopic level by TEM and atomic scale by NMR and mass spectrometry analyses. At pH 7.4, there was no significant change in particle size and morphology for up to 1 month, indicating the bulk erosion process. At the same time, the internal structure of the polymer conjugate was considerably altered. These data were consistent with the limited release of oligomeric species observed in the solution. Even if free ITA molecules were released, the bioconjugate remains only partially hydrolyzed.

## 3. Materials and Methods

### 3.1. Chemicals

All reagents and solvents were used without further purification, otherwise indicated.

For polymer synthesis: Diethyl ether was distilled from sodium/benzophenone ketyl. Toluene and dichloromethane were distilled from calcium hydride under a nitrogen atmosphere. All reactions involving air- or water-sensitive compounds were routinely conducted in glassware flame-dried under a positive pressure of nitrogen or argon. Itaconic anhydride, tin(II) 2-ethylhexanoate, and D,L-lactide were purchased from Sigma-Aldrich Chemical Co.

For polymer nanoparticle preparation and degradation: Salts were purchased from Sigma-Aldrich. Organic solvents were commercial analytical grade. Deionized (DI) water was taken from the PURELAB Ultra ELGA purification system. For the NMR experiments, the deuterated solvents were deuterium oxide (D_2_O) and deuterated trichloromethane from Innova-Chem, +99.8 atom D %.

### 3.2. Synthesis of the Two-Directional Polymer PLA-ITA

A solution of anhydrous 1,3-propanediol (freshly distilled over CaH_2_, 285 mg, 3.75 mmol) and D,L-lactide (10.0 g, 69.4 mmol) in anhydrous toluene (5 mL) was degassed using three freeze-thaw cycles. Tin(II) 2-ethylhexanoate (130 mg, 0.3 mmol) in toluene (0.2 mL) was added through a syringe, and the resulting mixture was placed in an oil bath at 140 °C. After 8 h, the obtained gel was cooled to room temperature, and itaconic anhydride (2.0 g, 17.8 mmol) was added. The reaction mixture was heated at 100 °C for 3 h. After cooling, the mixture was concentrated under reduced pressure. The residue was taken up into CH_2_Cl_2_ (150 mL) and washed with 0.1 N HCl (10 mL) and brine (10 mL). The organic phase was dried over MgSO_4_ and concentrated under reduced pressure. The residue was taken into CH_2_Cl_2_ (5 mL) and added dropwise into Et_2_O (100 mL). The solid was filtered and dried under a vacuum. The process was repeated to precipitate an acetone solution of the polymer into water. After drying under vacuum over P_2_O_5_, the title compound was obtained as a white solid (6.2 g).

### 3.3. Preparation of PLA-ITA Nanoparticles

Single emulsion: The organic phase was prepared by dissolving 60 mg of PLA-ITA conjugate polymer in 1.5 mL dichloromethane (DCM). The aqueous phase was polyvinyl alcohol (PVA) solution of 0.5 wt%. 4 mL of the aqueous phase was added to the organic phase, and immediately afterward, the resulting mixture was vortexed for 20 s. Subsequently, it was sonicated at 20% power for 1.5 min and then at 10% power for 30 s, using a sonication tip (Bandelin) dip inside the sample vial. The sample vial was placed in an ice bath to avoid heating the suspension due to sonication. The sample was stirred by magnetic stirring overnight at room temperature to evaporate the residual organic solvent. A sheet of laboratory paper was used to cover the vial from dust.

Nanoprecipitation: The organic phase was prepared by dissolving 10 mg PLA-ITA polymer in 0.75 mL acetone. The aqueous phase was deionized (DI) water. The organic phase, i.e., the polymer solution, was added dropwise (1 drop of roughly 10 μL per second) into 2 mL water under continuous gentle magnetic stirring. The sample was stirred overnight at room temperature to evaporate the residual organic solvent. Each batch was sonicated for about 10–15 s before combining all batches in a Falcon tube.

The NP suspension was stored in the fridge at −4 °C.

The concentration of the formulated PLA-ITA NPs was estimated from the weight of the polymer divided by the volume of DI water remaining after evaporation of the organic solvent. To determine the solvent volume inside the sample vial, one weighted the empty vial containing the magnetic stirrer beforehand, and weighted the sample vial containing NP suspension after overnight evaporation.

### 3.4. Degradation of PLA-ITA Nanoparticles

Briefly, the degradation of PLA-ITA nanoparticles (NPs) was tested in phosphate buffer saline (PBS) at pH 7.4 and 5.3 in DI water. After a given period, the suspension in PBS was separated into supernatant and pellet, named degraded supernatant and pellet. The obtained degraded supernatant was freeze-dried. Afterward, the freeze-dried precipitates were dissolved in deuterated water D_2_O; the obtained sample was measured by liquid NMR spectroscopy. 

Incubation of 4.5 mg mL^−1^ PLA-ITA NPs in PBS 10 mmol L^−1^ was carried out as follows. From the 5 mg mL^−1^ stock NPs aqueous suspension, 12.6 mL was pipetted into a Falcon tube 15 mL. Then, 1.4 mL of PBS 100 mmol L^−1^ at pH 7.4 or 5.3 was pipetted into this tube. The sample tube was closed, mixed, and placed in a rotator inside an oven set at 37 °C. After a given time, such as 1 month and 2 months, roughly half of the incubated sample tube was transferred to another 15 mL Falcon tube to avoid centrifuging a full tube. These two tubes were centrifuged at 10,000 RCF (relative centrifugal force) for 15 min. The supernatant from two tubes was put in another tube. The pellet was dried at room temperature. The degraded supernatant was freeze-dried overnight using a Christ Alpha 2–4 LSCbasic lyophilizer. One tried to dissolve the freeze-dried precipitate in 600 µL D_2_O; the solution was transferred to an NMR tube.

PBS 100 mmol L^−1^ in H_2_O was prepared as follows. Stock A was NaH_2_PO_4_ solution of 0.1 mol L^−1,^ and stock B was KH_2_PO_4_ 0.1 mol L^−1^. Both these salts quickly dissolved in DI water. To 40 mL of stock A and stock B, 0.32 g of NaCl was added and dissolved. Different proportions of stock A and stock B were mixed to obtain different pH. 20 mL PBS 100 mM with pH 7.4 and 5.3 was comprised of 4 mL stock A and 16 mL stock B, and 19.5 mL stock A and 0.5 mL stock B, respectively. The pH of PBS 100 and 10 mmol L^−1^ was checked by a digital pH meter.

### 3.5. Characterization Methods

For characterizing the synthesized PLA-ITA, IR spectra of solid or neat liquid were obtained on a Fourier Transform Shimadzu IRAffinity-1 spectrometer. Only significant absorptions are listed. The ^1^H and ^13^C NMR spectra were acquired on Bruker Avance 300 (300 MHz and 75 MHz, for ^1^H and ^13^C, respectively) spectrometers. Recognition of methyl, methylene, methine, and quaternary carbon nuclei in ^13^C NMR spectra was based on the J-modulated spin-echo sequence.

The size and colloidal stability of NPs suspension were studied by Dynamic Light Scattering (DLS) and Zeta-potential (ZP) measurements (Zetasizer Nano ZS90, Malvern Panalytical, Malvern, The United Kingdom). Samples of 0.15 mg/mL of NPs were prepared in DI water for DLS or in 1 mM KCl for ZP measurements. pH measurements were conducted on a Hanna Instruments edge^®^ pH digital meter.

Morphology and size of NPs were observed by TEM under a 120 kV and 80 kV JEOL JEM-1400 microscope. NP suspensions were deposited on copper grids and observed with staining. For the original polymer NPs, negative staining was done with phosphotungstic acid. For the polymer NPs degraded at pH 7.4, after negative staining with phosphotungstic acid, the TEM grid was washed twice with water to remove the residual salts coming from the degradation medium. For the polymer NPs degraded at pH 5.3, the TEM grid was washed twice with water prior to negative staining with uranyl acetate to prevent precipitating uranyl acetate in phosphate. Negative staining of these acid-degraded samples with phosphotungstic acid (pH 7.4) did not work, probably due to incompatible pH. Then the TEM grid was washed twice with water again to remove the residual salts.

Solution spectra for the degradation study were recorded at 298 K. ^1^H, and ^13^C NMR measurements were acquired with a Bruker Avance 400 MHz spectrometer equipped with a 5 mm BBI probe head and operated at a magnetic field strength of 9.4 T, respectively. D_2_O or DCCl_3_ were used as deuterated solvents. Typically, ^1^H spectra were recorded with one pulse sequence at a 30° flip angle (pulse duration 2.8 μs), using 2 s recycle delay, 3 s acquisition time, and 128 scans. ^1^H{^1^H} COSY, ^1^H{^13^C} HSQC and ^1^H{^13^C} HMBC spectra were carried out on some selected samples using standard Bruker pulse sequences. The recycle period was shortened to 1 s for all these 2D experiments. Typically, mixing times of 17 ms and 133 ms corresponding to ¼J_H-C_ (^1^J_H-C_ = 145 Hz) and ½J_H-C_ (^2^J_H-C_ = 7.5 Hz) were employed for ^1^H{^13^C} HSQC and ^1^H{^13^C} HMBC experiments, respectively. The ^13^C spectra were obtained with a standard power-gated decoupling pulse sequence, using typically 1 s recycle delay, 1.2 s acquisition time, and 25,600 scans. J-modulated spin-echo sequence has also been used to identify methyl, methylene, methine, and quaternary carbon nuclei in ^13^C NMR spectra. According to conventional standards, chemical shifts are reported relative to 1% Me_4_Si in CDCl_3_ for both ^1^H and ^13^C.

Quantitative ^1^H NMR spectroscopy was based on the calibration of itaconic acid and lactic acid solution in D_2_O with known concentrations. 

ESI (electrospray ionization) mass spectra were recorded on an LTQ-Velos Pro Thermofisher Scientific spectrometer, and MALDI-TOF (Matrix-assisted laser desorption-ionization time of flight) mass spectra were recorded in an UltrafleXtreme apparatus (Bruker Daltonics, Billerica, MA, the US) using the service from Small Molecule Mass Spectrometry platform of ICSN (Centre de Recherche de Gif—http://icsn.cnrs.fr (accessed on 18 November 2022)). The ESI MS and MALDI MS measurements were performed in negative and positive ion modes, respectively.

For ESI MS measurement of the water-soluble degradation products, the residual phosphate salts in the pellet had to be removed. Specifically, after freeze-drying the degraded supernatant, the acquired powder was sonicated in 2 mL methanol for 20 min. Then, the sample is allowed to stand for 1 h before centrifuging 10,000 RCF for 5 min and took out the supernatant. This liquid was evaporated under a fume hood for 2 nights, leaving the solid suitable for ESI MS measurement.

## 4. Conclusions

A novel synthetic approach is presented to produce PLA-ITA polymer conjugate NPs, serving as a drug delivery system. The NP degradation products in PBS at pH 7.4 were monitored by ^1^H and ^13^C NMR spectroscopy to analyze the nature of the species released in the solution. Using two-dimensional correlation experiments (COSY, HSQC, and HMBC), a series of small soluble oligomers were identified and quantified. The primary water-soluble degradation products were found to correspond to seven molecules based on ITA and LA, including the monomers LA and ITA, the dimer (LA)_2_, and the mixed coupled molecules LA-ITA, ITA-LA, (LA)_2_-ITA, and ITA-(LA)_2_. ESI(-) mass spectrometry also detected mixed ITA-LA derivatives. Moreover, MALDI-TOF mass spectrometry was employed to characterize the insoluble degraded polymer residues, revealing the fragmentation of the initial NPs into smaller ones. Quantitative analysis showed that the degradation process is slow and that drug release remains limited. TEM images revealed conservation of particle size during degradation, which is relatively consistent with a bulk erosion mechanism and limited species release. 

In the subsequent studies, to improve the drug delivery efficiency of this polymer-ITA conjugate NPs, it appears necessary to employ polymers that degrade faster than D,L-PLA. For example, one could explore a copolymer of lactic acid and glycolic acid PLGA with a low ratio of lactic acid to glycolic acid or tune the crystallinity and molar mass of PLA.

## Figures and Tables

**Table 1:** Assignment of ESI(-) mass spectra of water-soluble degradation products of PLA-ITA NPs after 2-month incubation in PBS at pH 7.4.

Species	Chemical Structure and Formula	*m*/*z* of Negative Ion [M-H]
LA	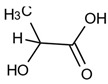 C_3_H_4_O_2_	89.3
ITA	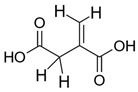 C_5_H_6_O_4_	129.1
ITA-propane-2-diol	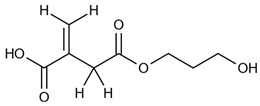 C_8_H_12_O_5_	187.1
ITA-LA	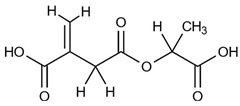 C_8_H_10_O_6_	201.1

**Table 2:** Assignment of positive mode MALDI mass spectra of original and degraded PLA-ITA pellets. Incubation in PBS at pH 5.3 was done for 1 month.

Species	MS Peak Mode	*m*/*z*	Assignment	
Original PLA-ITA	[M + Na]^+^ (*n* = *x* + *y*)	3131.89	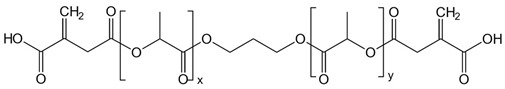	*n* = 39
		3203.91		*n* = 40
		3275.93		*n* = 41
		3164.14	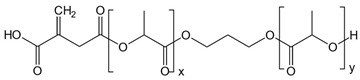	*n* = 41
		3308.23		*n* = 43
Degraded PLA-ITA	[M + Na]^+^ (*n* = *x* + *y*)	1867.62	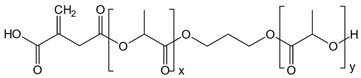	*n* = 23
		1939.64		*n* = 24
	[M + K]^+^	1881.58	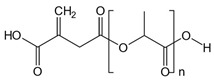	*n* = 24
		1953.61		*n* = 25

**Table 3:** Itaconate (ITA) and lactate (LA) release after PLA-ITA NPs degradation as analyzed by quantitative ^1^H NMR spectroscopy.

PLA-ITA NPs Degradation	% ITA Release		% LA Release	
	1 Month	2 Months	1 Month	2 Months
pH 7.4	18.4	17.4	8.7	8.8
pH 5.3	7.7	16.0	2.8	6.7

## Data Availability

Not applicable.

## Supplementary Materials

The following supporting information can be downloaded at: https://www.mdpi.com/article/10.3390/ijms232214461/s1.
