# Supplementary material for: Degradation of Polymer-Drug Conjugate Nanoparticles Based on Lactic and Itaconic Acid
Source: Int J Mol Sci. 2022 Nov 21;23(22):14461. doi: 10.3390/ijms232214461 (PMC9699510; doi:10.3390/ijms232214461)
Supplement: Supplementary file 1 [file ijms-23-14461-s001.zip › ijms-2000358-supplementary.pdf]

Supporting Information

1. Spectroscopic data of PLA-ITA polymer

From the integration ratio between lactate and itaconate ^{1}H NMR peaks, the molecular weight of PLA-ITA was estimated as M_n = 3850-4050 g.mol^{-1}.

The peaks of IR, NMR, and MS are listed as follows:

- IR (neat, cm^{-1}) ν: 2995, 1755, 1456, 1382, 1359, 1211, 1182, 1130, 1087, 4043, 871;
- ^{1}H NMR (300 MHz, $CDCl_3$)

Chemical shift (ppm)	Multiplicity	Number of protons	J coupling (Hz)	Assignment
6.45	singlet,	2H		H_2C=
5.88	singlet	2H		H_2C=
5.17	quadruplet	52H	7.2	$OC\mathit{H}(CH_3)CO_2$
4.20	triplet	4H	6.3	$OCOC\mathit{H}_2CH_2C\mathit{H}_2OCO$
3.45	doublet	2H	16.8	$(CH_2{=}C)C\mathit{H}_2CO_2H$
3.38	doublet	2H	16.8	$(CH_2{=}C)C\mathit{H}_2CO_2H$
1.99	quintuplet	2H	6.3	$OCOCH_2C\mathit{H}_2CH_2OCO$
1.57	doublet	147H	7.2	$OCH(C\mathit{H}_3)CO_2$
1.50	doublet	3H	7.2	$OCH(C\mathit{H}_3)CO_2H$

- ^{13}C NMR (100 MHz, $CDCl_3$):

Chemical shift (ppm)	Assignment
170.1-169.7 (m[a])	COO
132.8	C in $H_2C{=}C$
130.9	CH_2 in $H_2C{=}C$
69.6-69.1 (m)	CH in $OCH(CH_3)CO_2$
61.8	CH_2 in $OCOCH_2CH_2CH_2OCO$
36.9	CH_2 in $(CH_2{=}C)CH_2CO_2H$
27.9	CH_2 in $OCOCH_2CH_2CH_2OCO$
16.76	CH_3 in $OCH(CH_3)CO_2$

[a] m means multiplet

- MS (ESI-) m/z = [328.1 + $(36.03)_n$] with n = 3 to 50 for $[C_{13}H_{14}O_8 + (C_3H_4O_2)_n]^{2-}$.

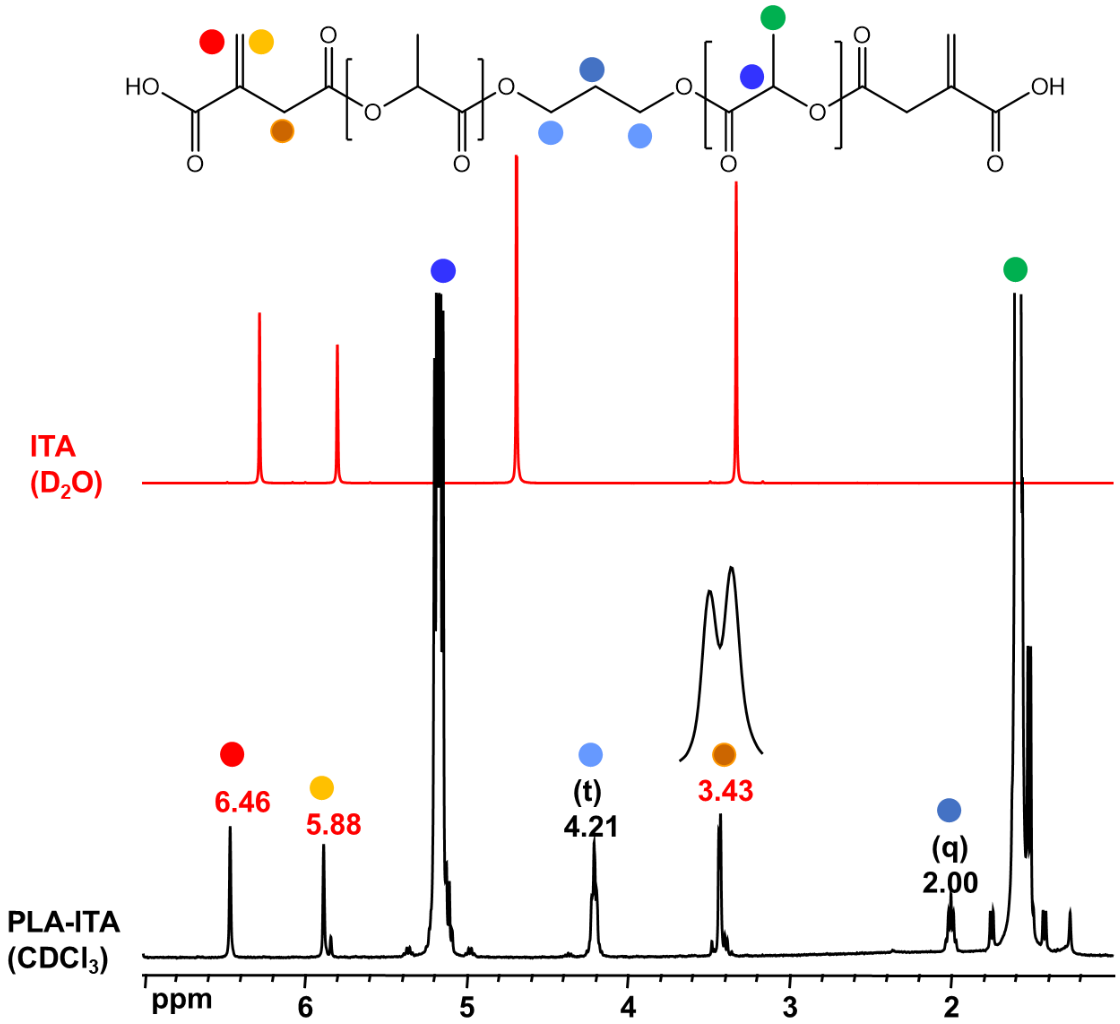

Figure S1. ^{1}H NMR spectrum (in $CDCl_3$) of the itaconate-PLA polymer compared to the spectrum of itaconic acid (in D_2O).

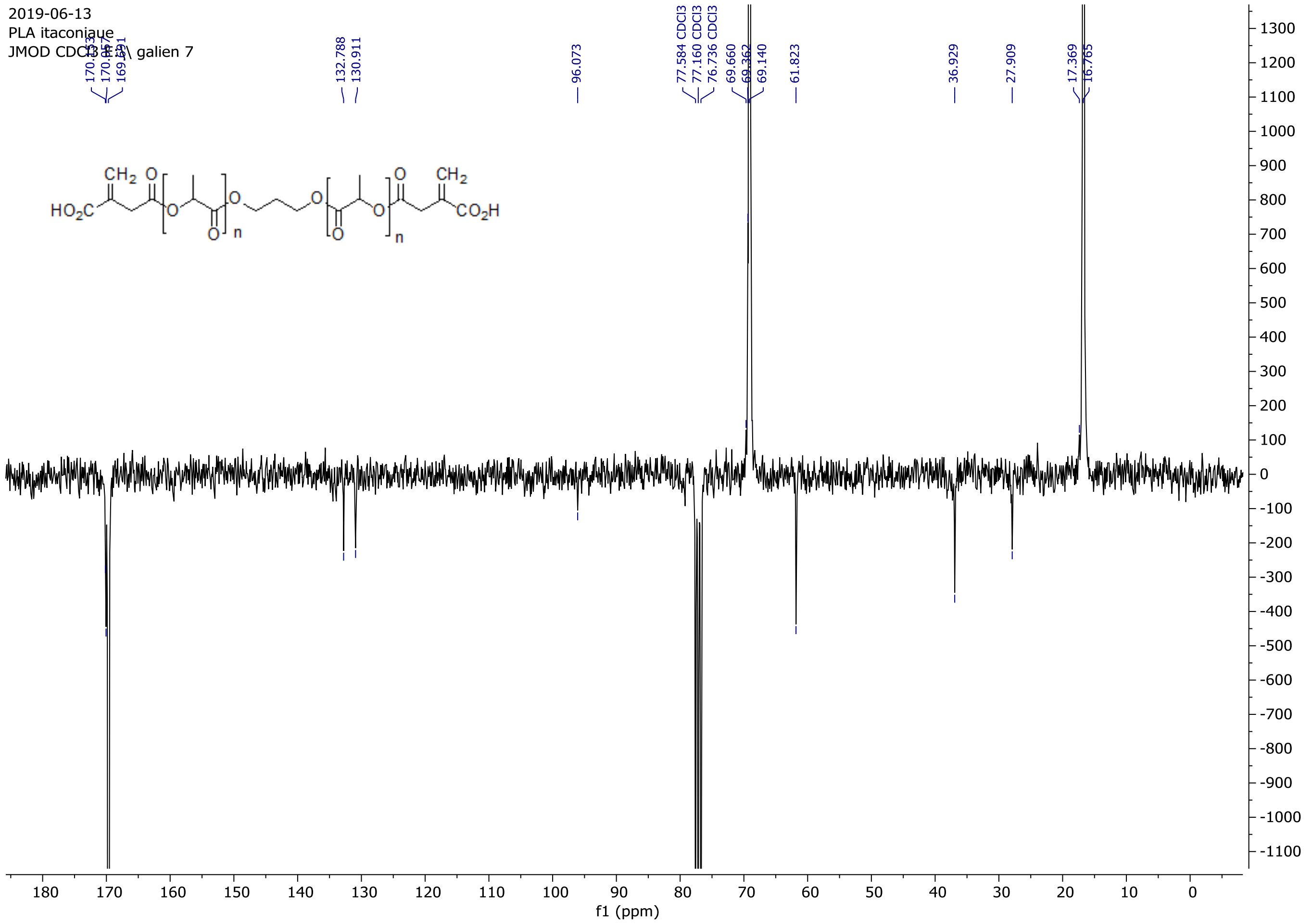

Figure S2. ^{13}C DEPT-135 NMR spectrum (in $CDCl_3$) of the itaconate-PLA polymer.

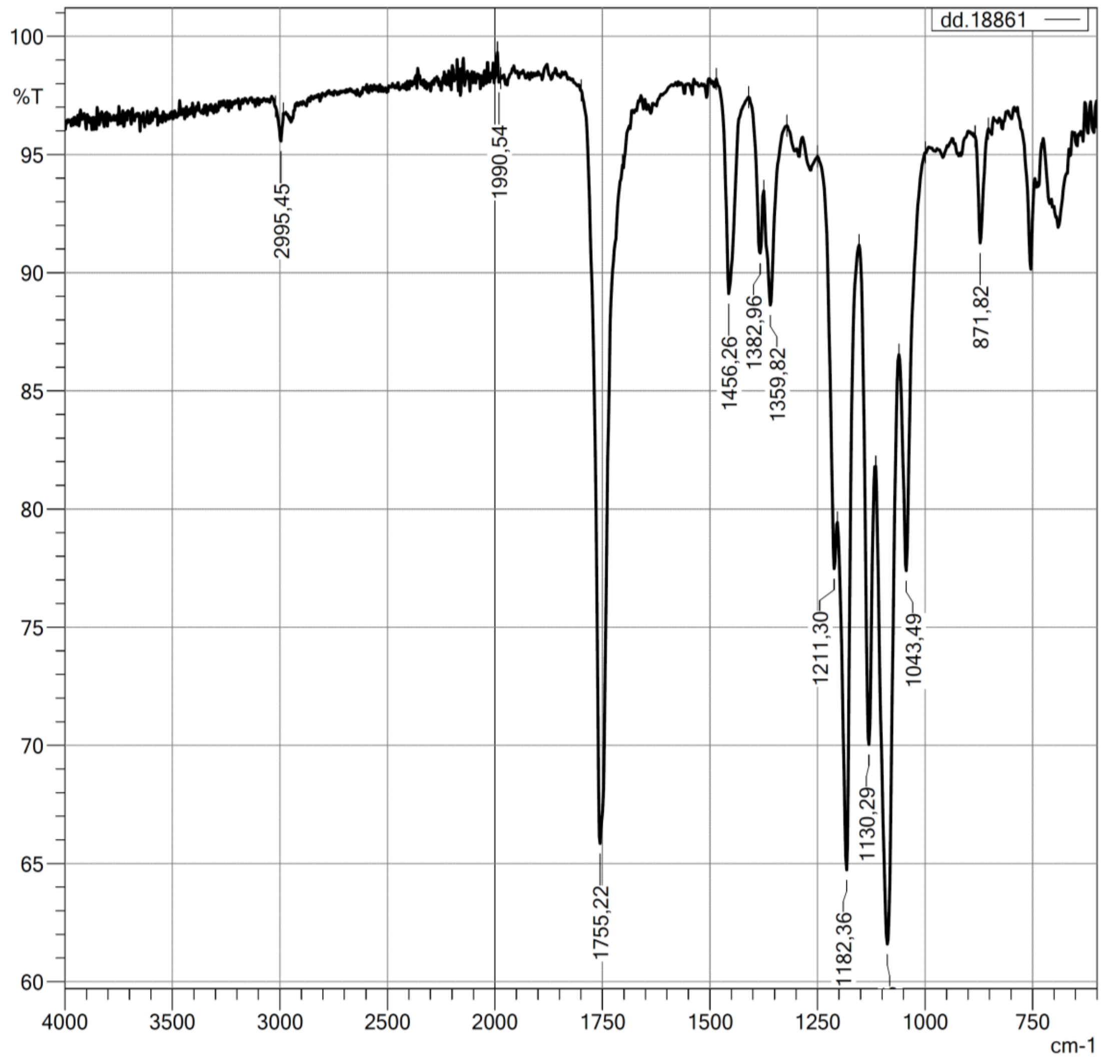

Figure S3. IR spectrum of the itaconate-PLA polymer.

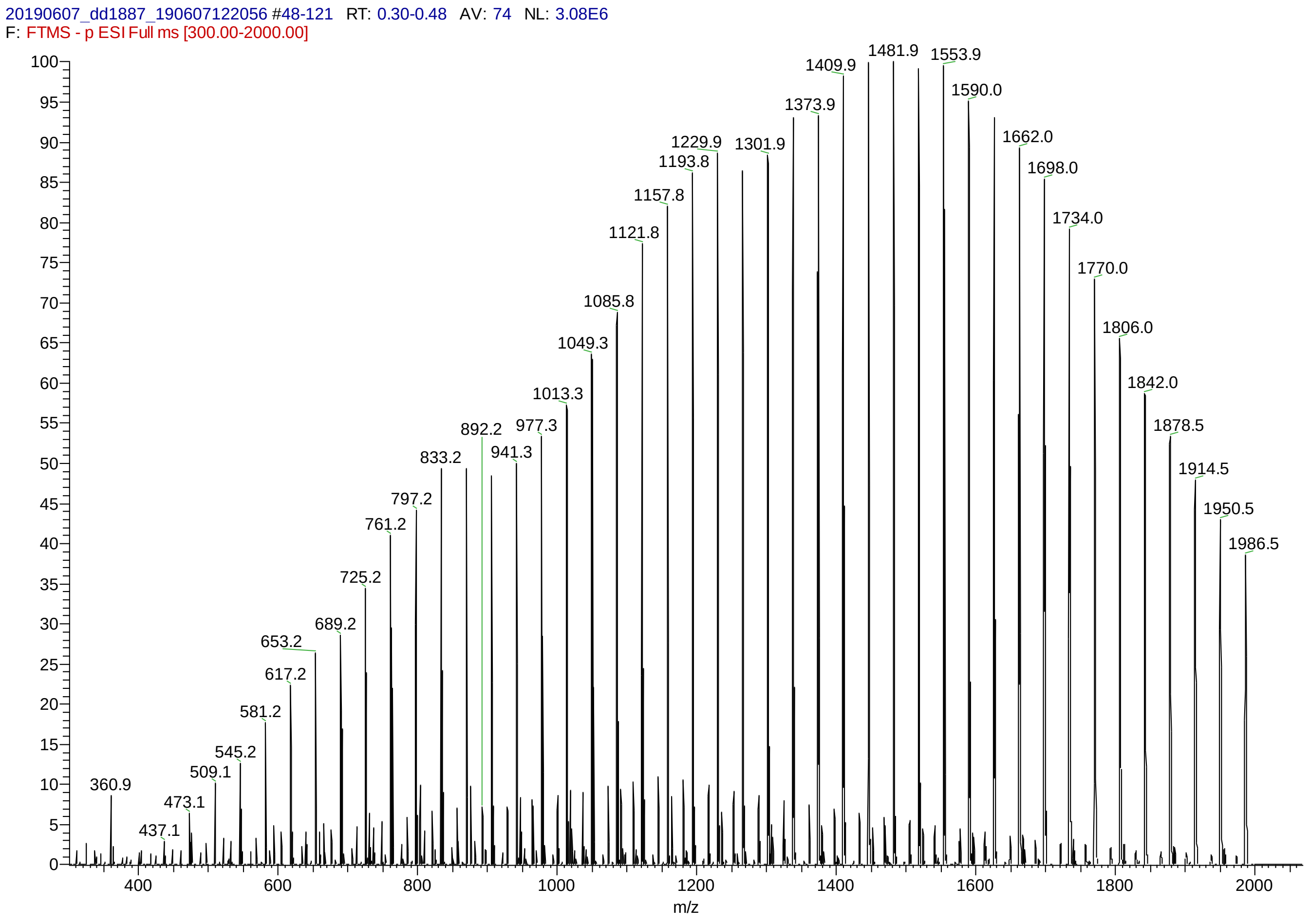

Figure S4. ESI(-) mass spectrum of the itaconate-PLA polymer in MeOH/DCM.

2. Preparation of PLA-ITA polymer-drug conjugate nanoparticles

Table S2. Mean hydrodynamic diameter (z_average) and polydispersity value of several PLA-ITA NPs formulations

Method	[PLA-ITA] (mg.mL^{-1})	Organic and aqueous phase	z_average (nm)	PdI	Observation
Single emulsion	5	1.5 mL dichloromethane, 4 mL PVA 4 wt% aqueous solution	301 ± 12	0.13 ± 0.02	
	10		310 ± 7	0.12 ± 0.09	
	15		331 ± 12	0.05 ± 0.03	
	20		323 ± 14	0.07 ± 0.01	some precipitate after solvent evaporation
Nanoprecipitation	5, 10, 15, 20	1.5 mL acetone, 4 mL PVA 4 wt% aqueous solution	aggregate		
Nanoprecipitation small volume	5	0.75 mL acetone, 2 mL DI water	343 ± 36	0.49 ± 0.07	

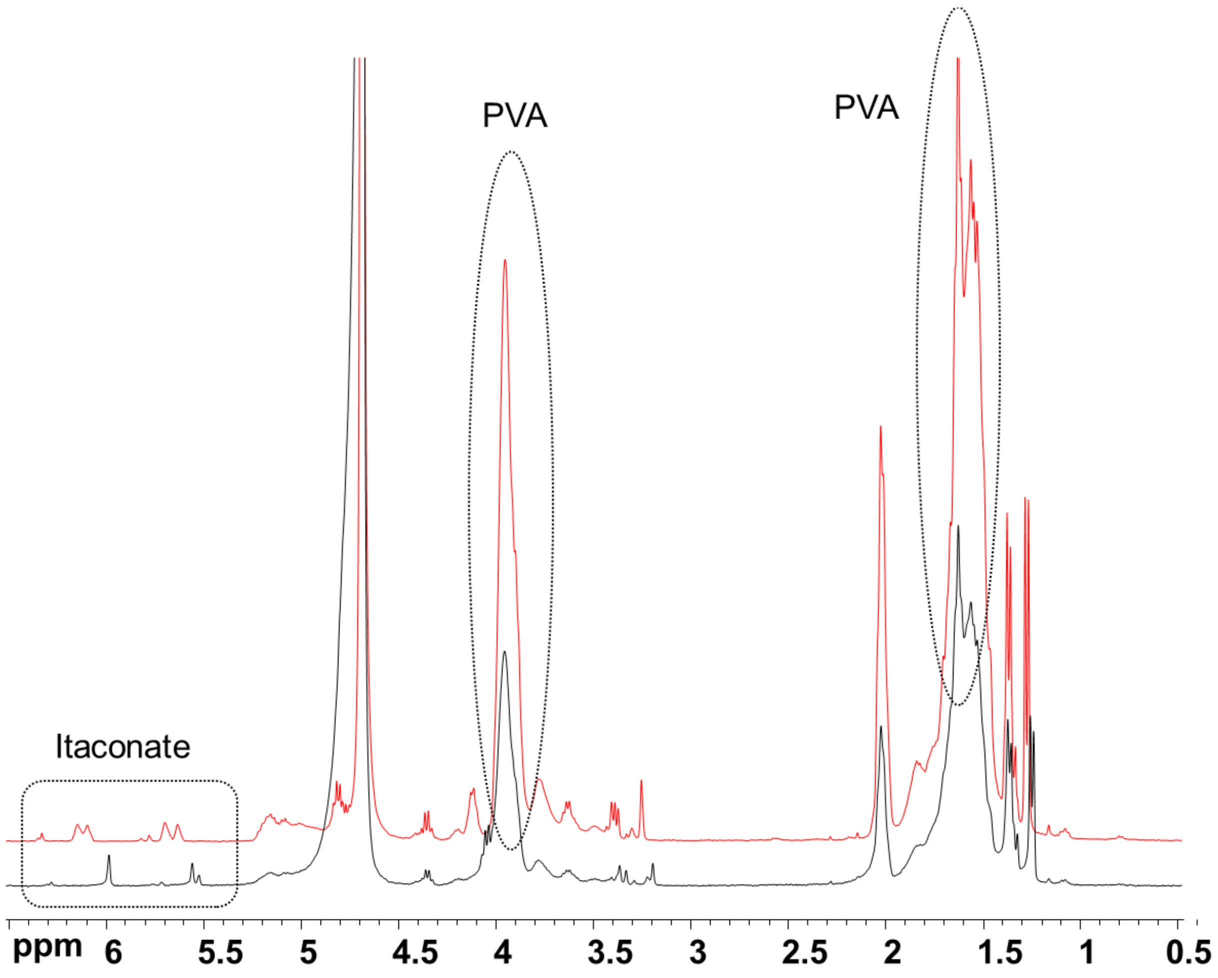

Figure S5. ^{1}H NMR spectra of water-soluble degradation products of PLA-ITA single emulsion degraded in pH 7.4 and 5.3 for 1 month 11 days.

3. Identification of degradation products of PLA-ITA nanoparticles

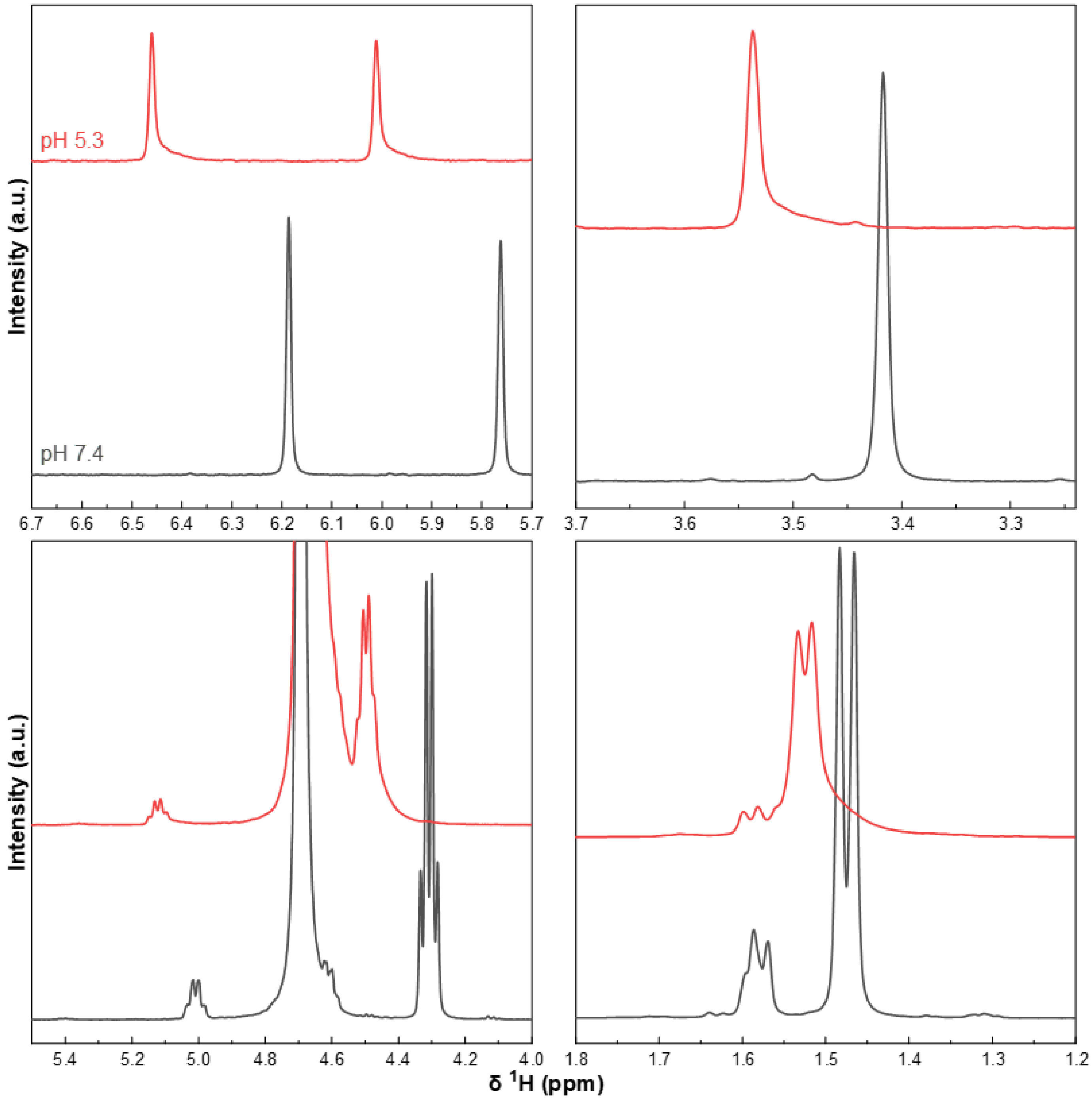

Figure S6. Zoom in ^{1}H NMR spectra of itaconic acid and lactic acid solution mixture incubated at pH 5.3 and 7.4 for 1 month.

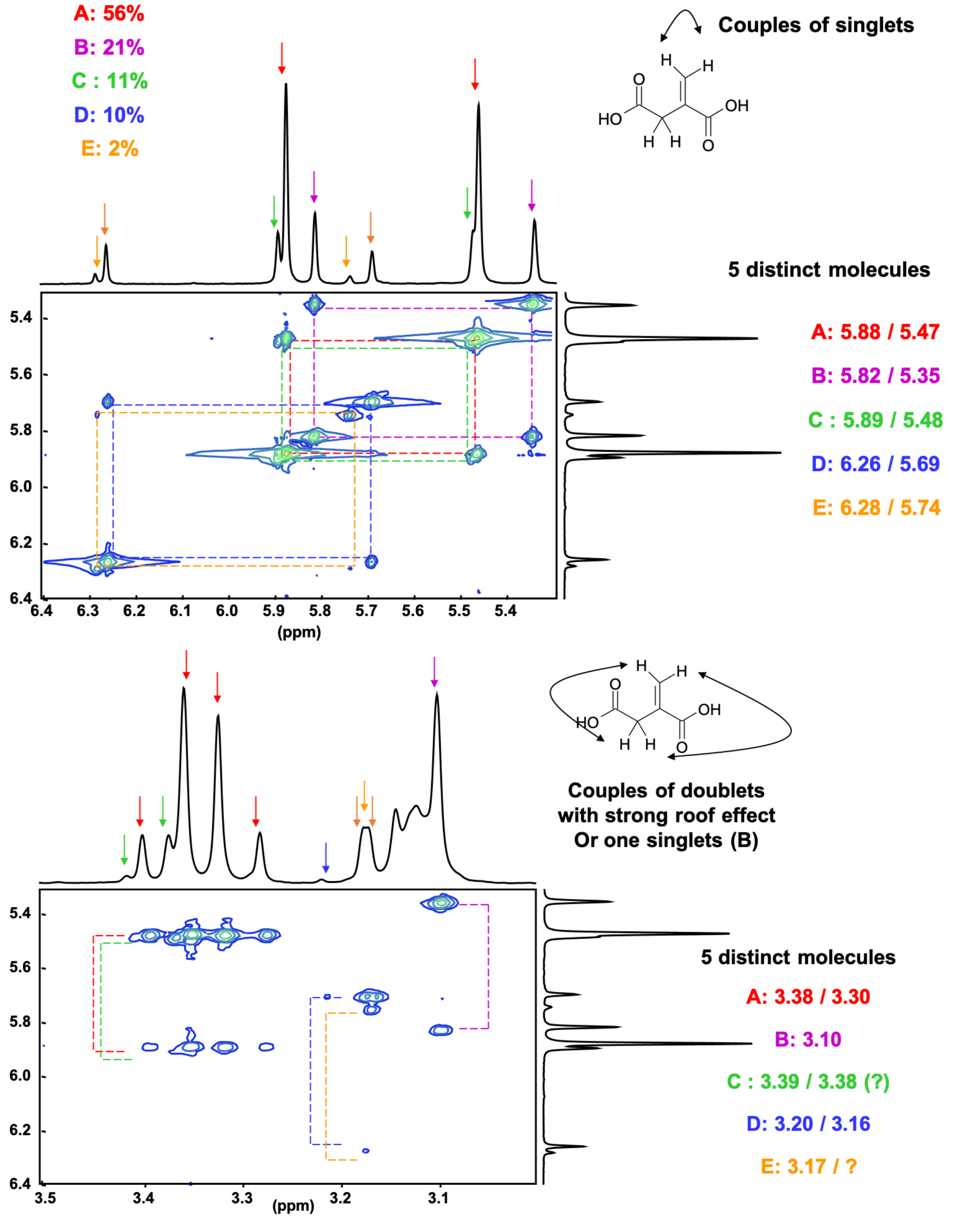

Figure S7. Zoom on ITA resonances in the ^{1}H-^{1}H COSY NMR spectrum of water-soluble degradation products of PLA-ITA nanoprecipitation degraded at pH 7.4 for 2 months.

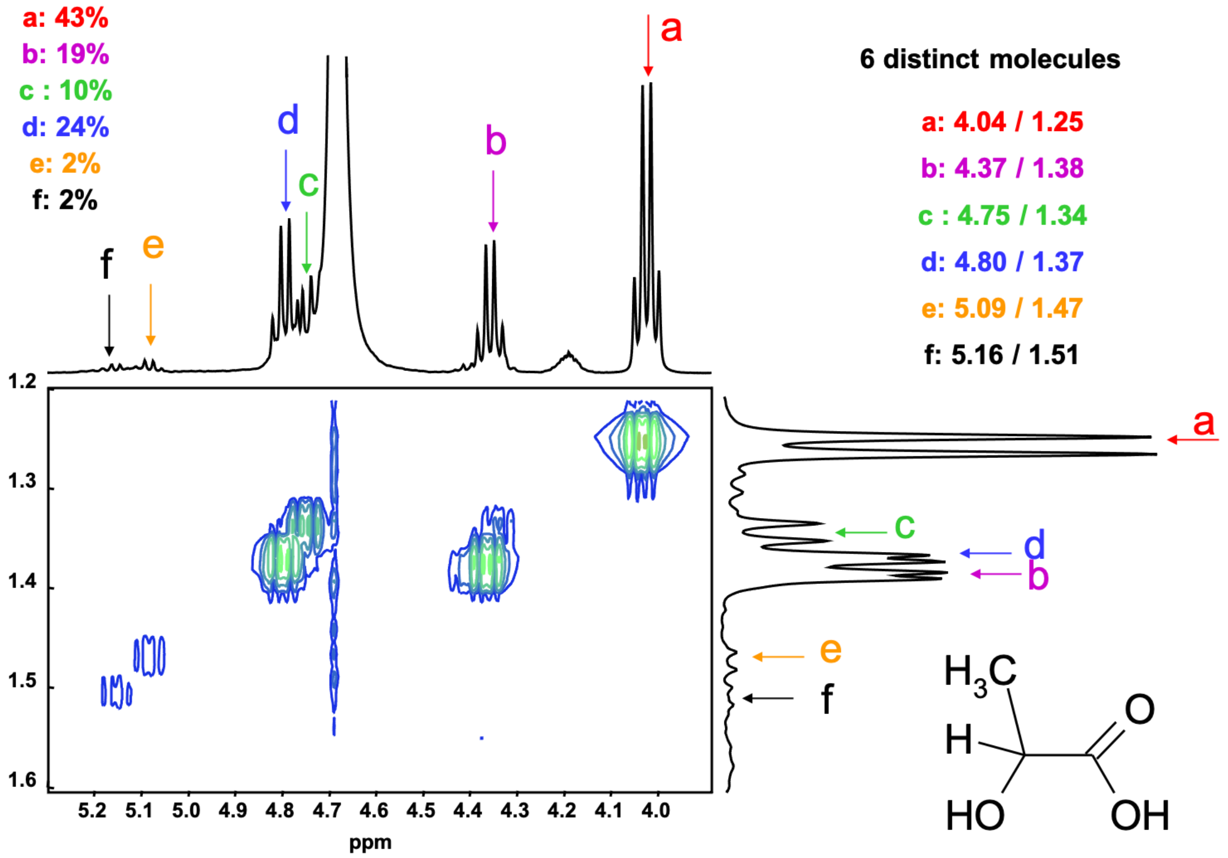

Figure S8. Zoom on LA resonances in the ^{1}H-^{1}H COSY NMR spectrum of water-soluble degradation products of PLA-ITA nanoprecipitation degraded at pH 7.4 for 2 months.

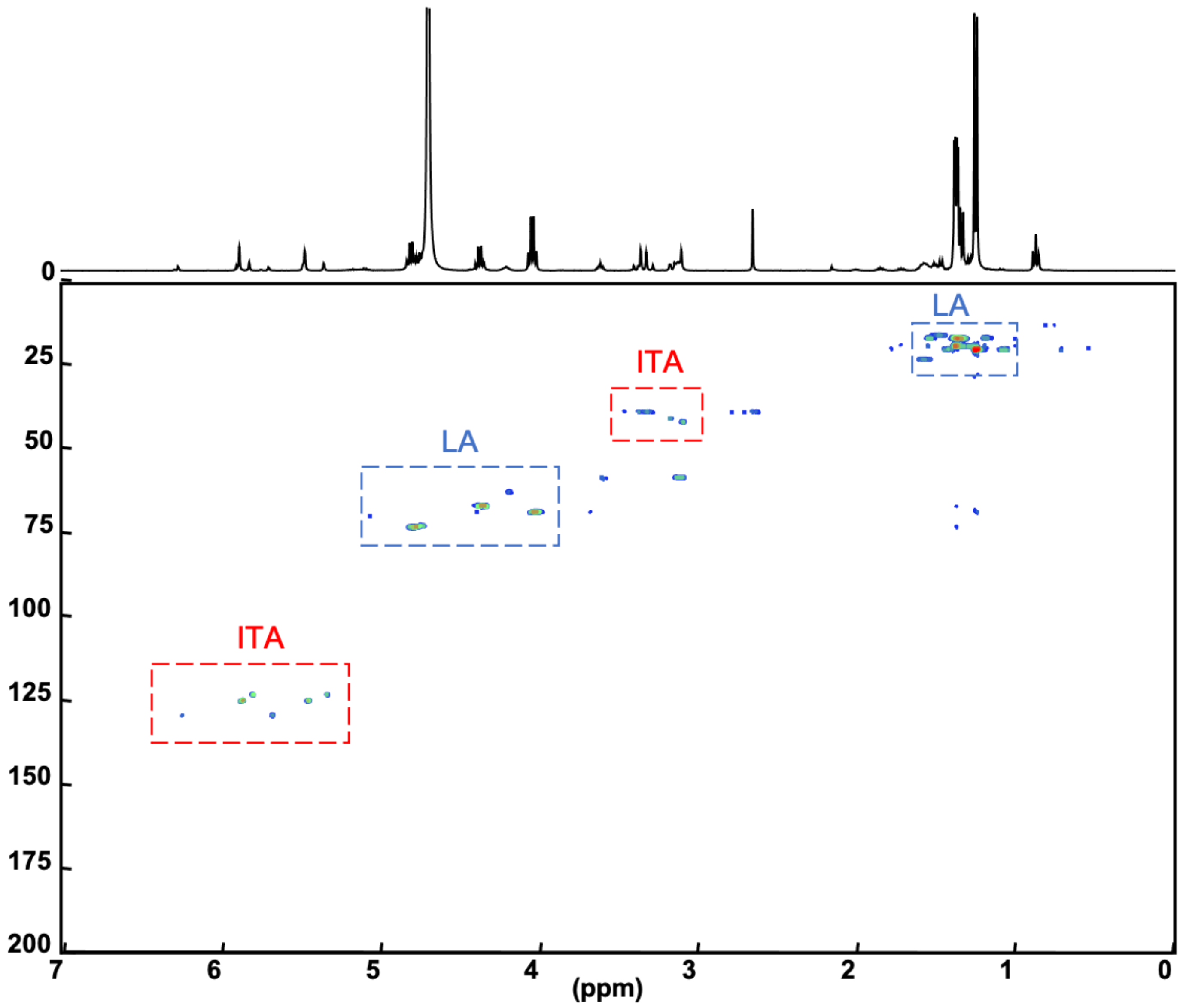

Figure S9. ^{1}H-^{13}C HSQC NMR spectra of water-soluble degradation products of PLA-ITA nanoprecipitation degraded at pH 7.4 for 2 months.

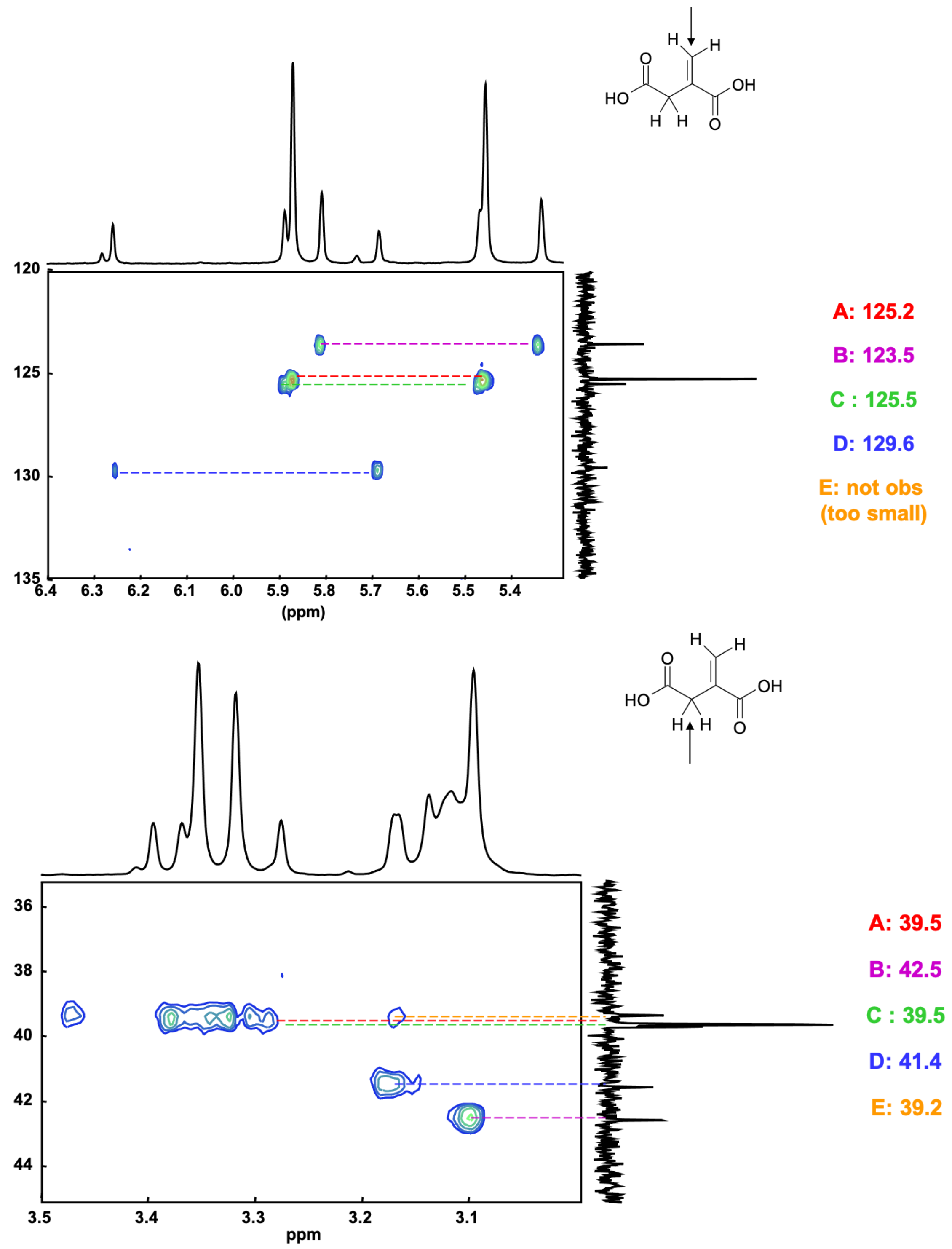

Figure S10. Zoom on ITA resonances in the ^{1}H-^{13}C HSQC NMR spectra of water-soluble degradation products of PLA-ITA nanoprecipitation degraded at pH 7.4 for 2 months.

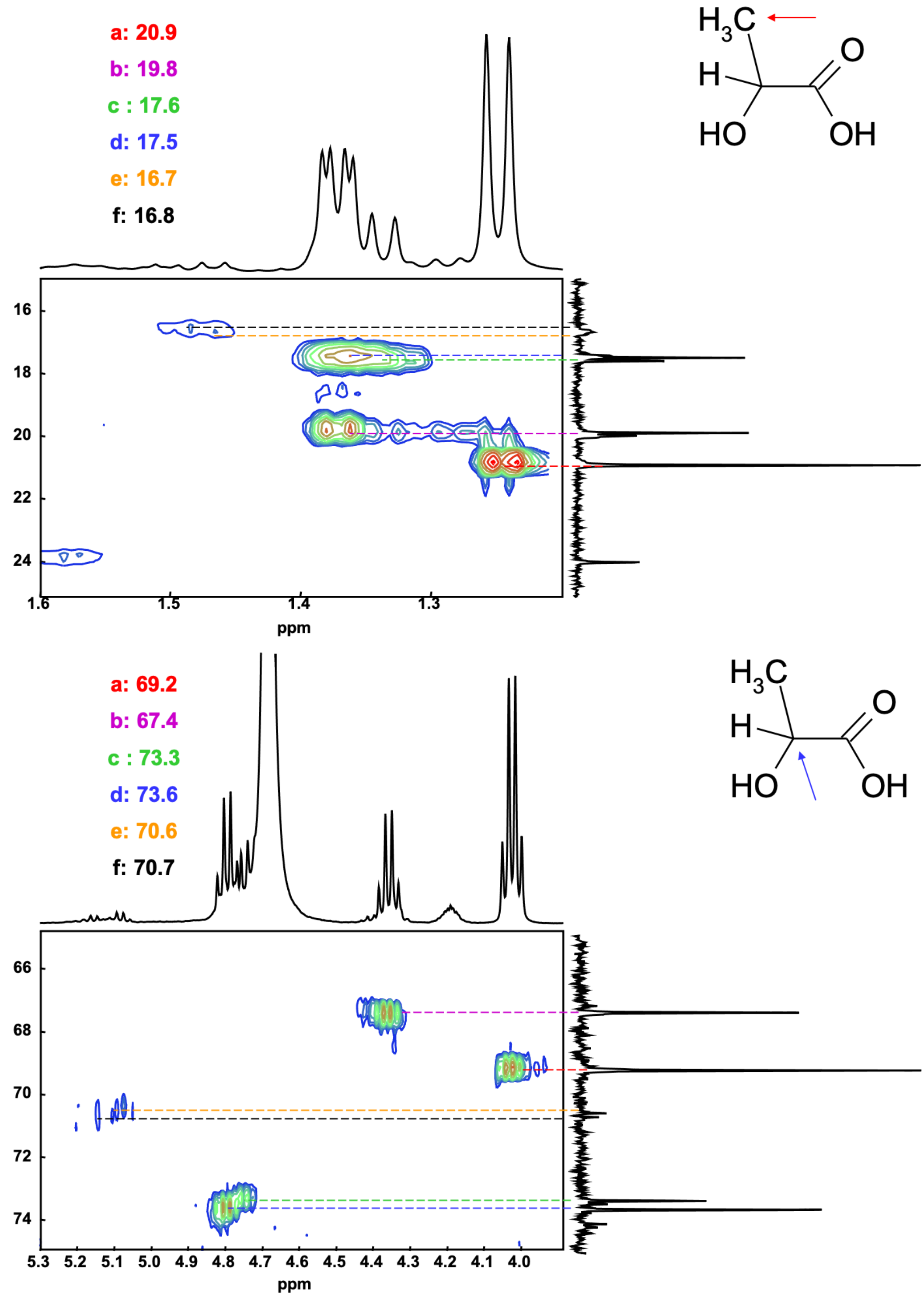

Figure S11. Zoom on LA resonances in the ^{1}H-^{13}C HSQC NMR spectra of water-soluble degradation products of PLA-ITA nanoprecipitation degraded at pH 7.4 for 2 months.

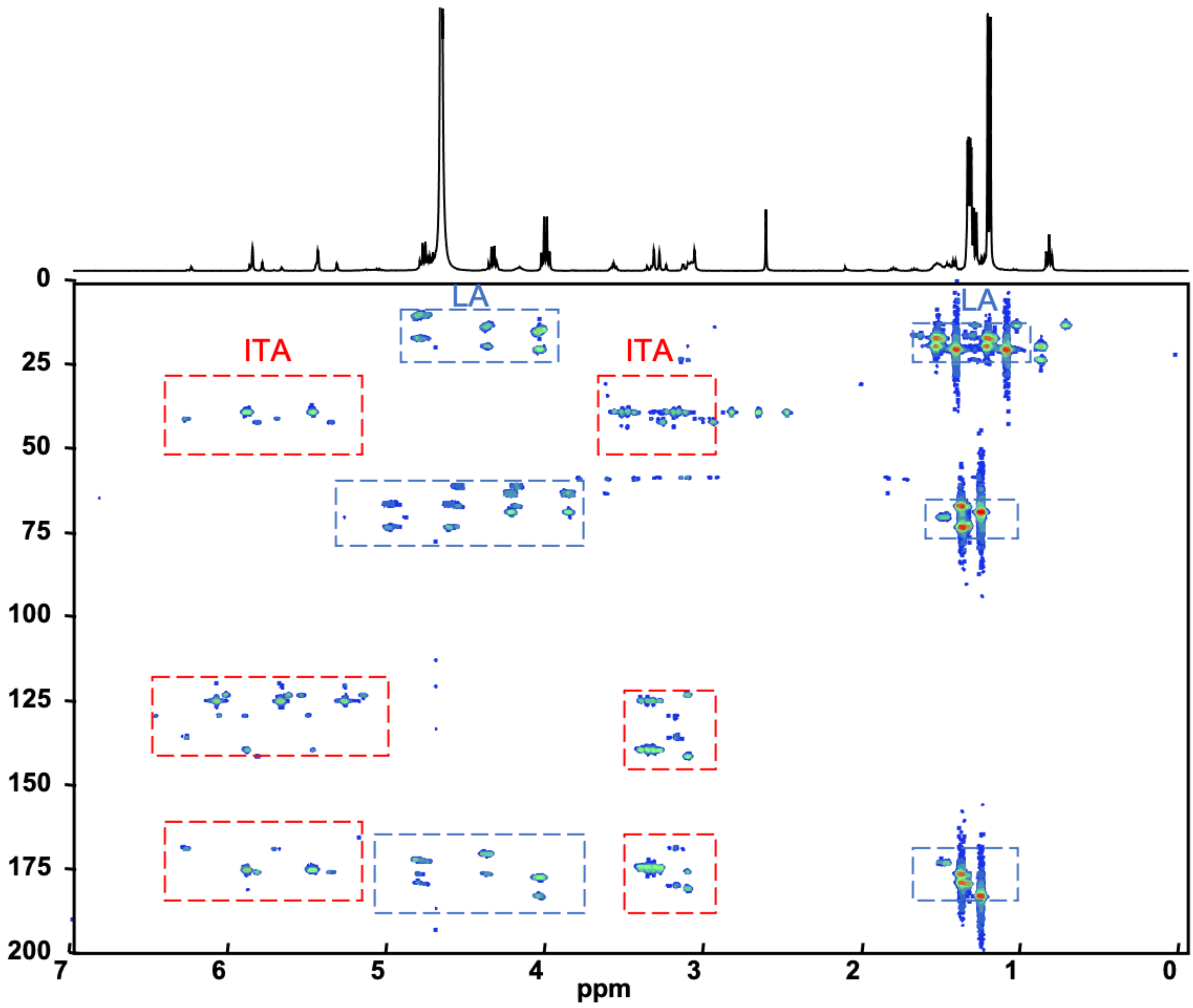

Figure S12. ^{1}H-^{13}C HMBC NMR spectra of water-soluble degradation products of PLA-ITA nanoprecipitation degraded at pH 7.4 for 2 months.

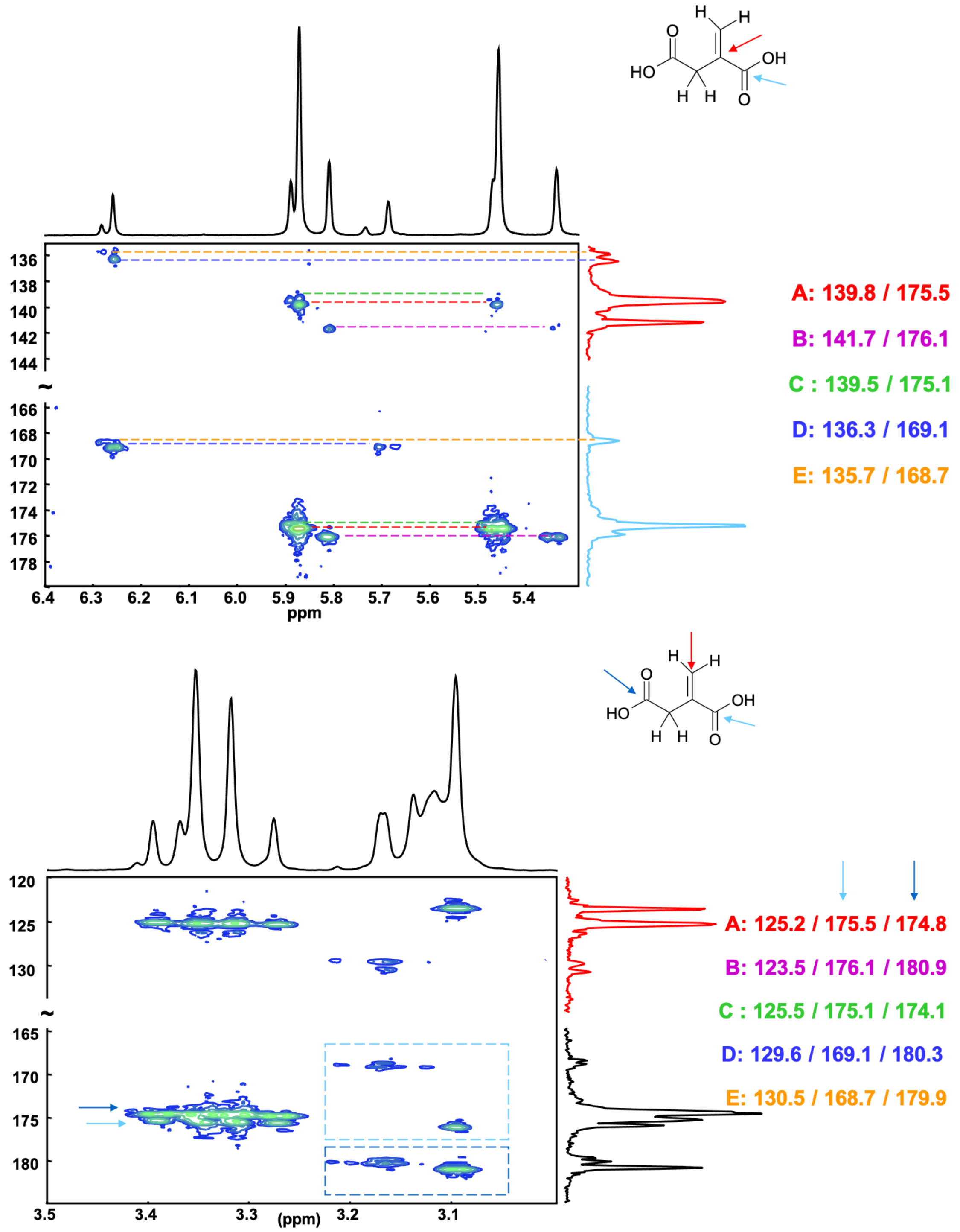

Figure S13. Zoom on ITA resonances in the ^{1}H-^{13}C HMBC NMR spectra of water-soluble degradation products of PLA-ITA nanoprecipitation degraded at pH 7.4 for 2 months.

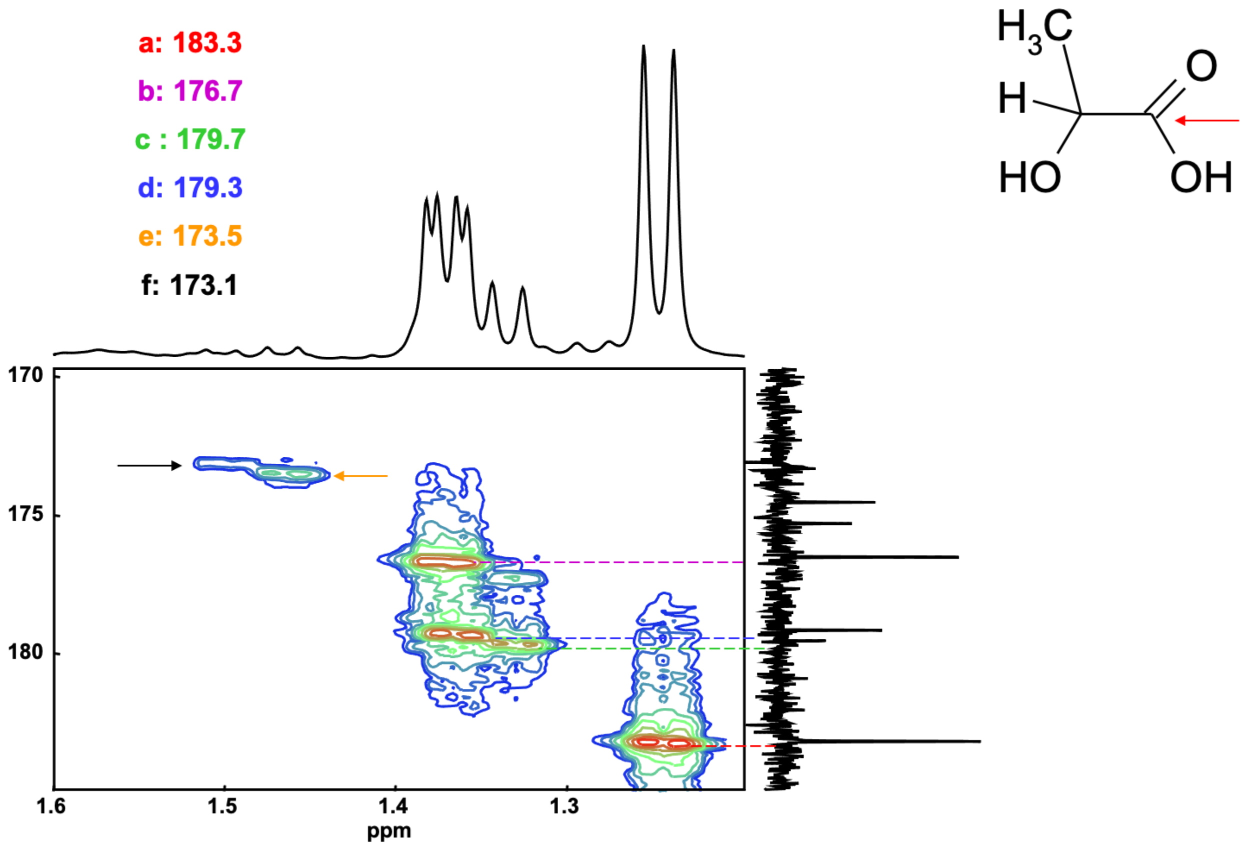

Figure S14. Zoom on LA resonances in the ^{1}H-^{13}C HMBC NMR spectra of water-soluble degradation products of PLA-ITA nanoprecipitation degraded at pH 7.4 for 2 months.

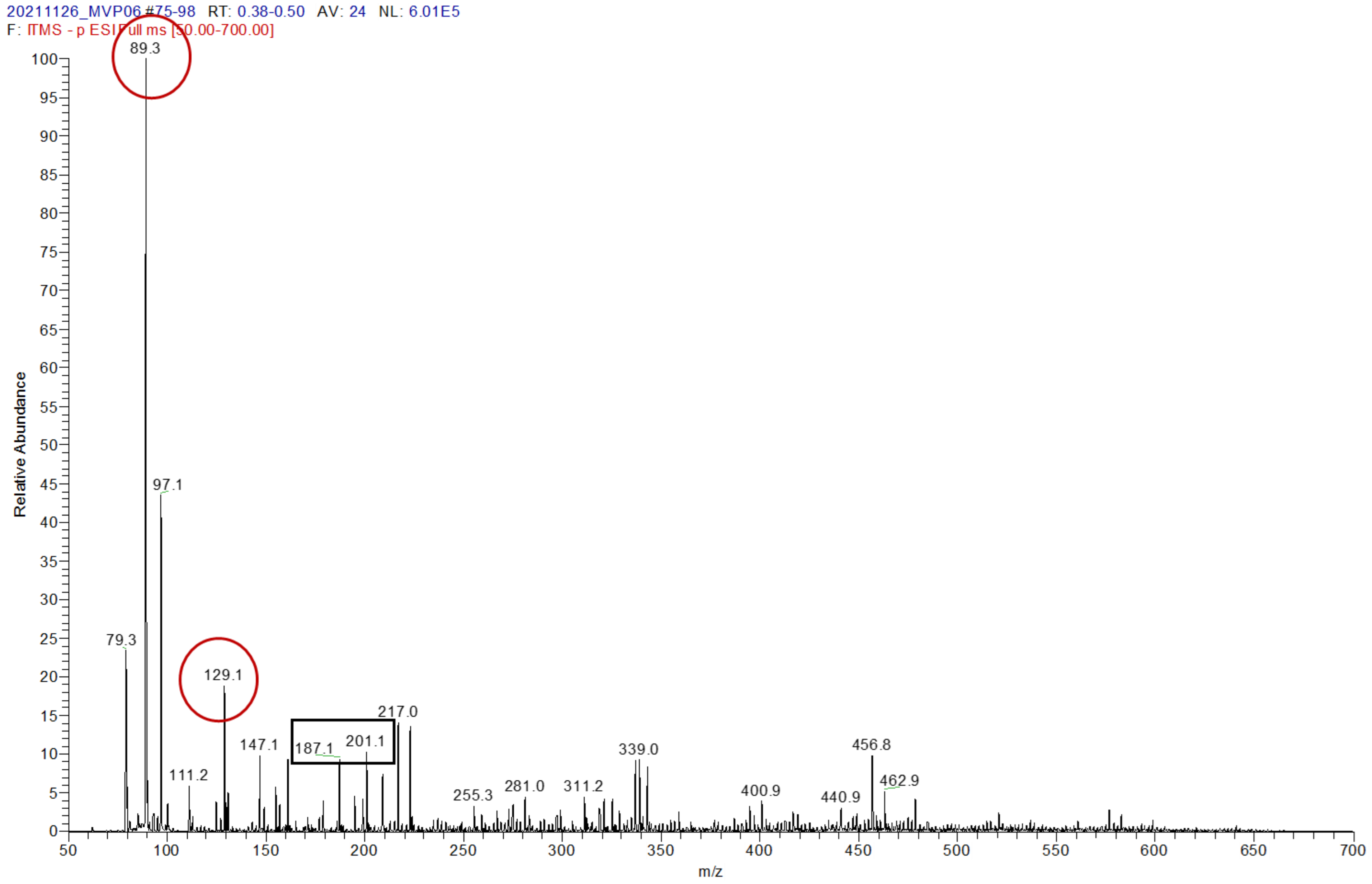

Figure S15. ESI(-) Mass spectrum of water-soluble degradation products of PLA-ITA nanoprecipitation obtained after degradation in PBS at pH 7.4 for 2 months.

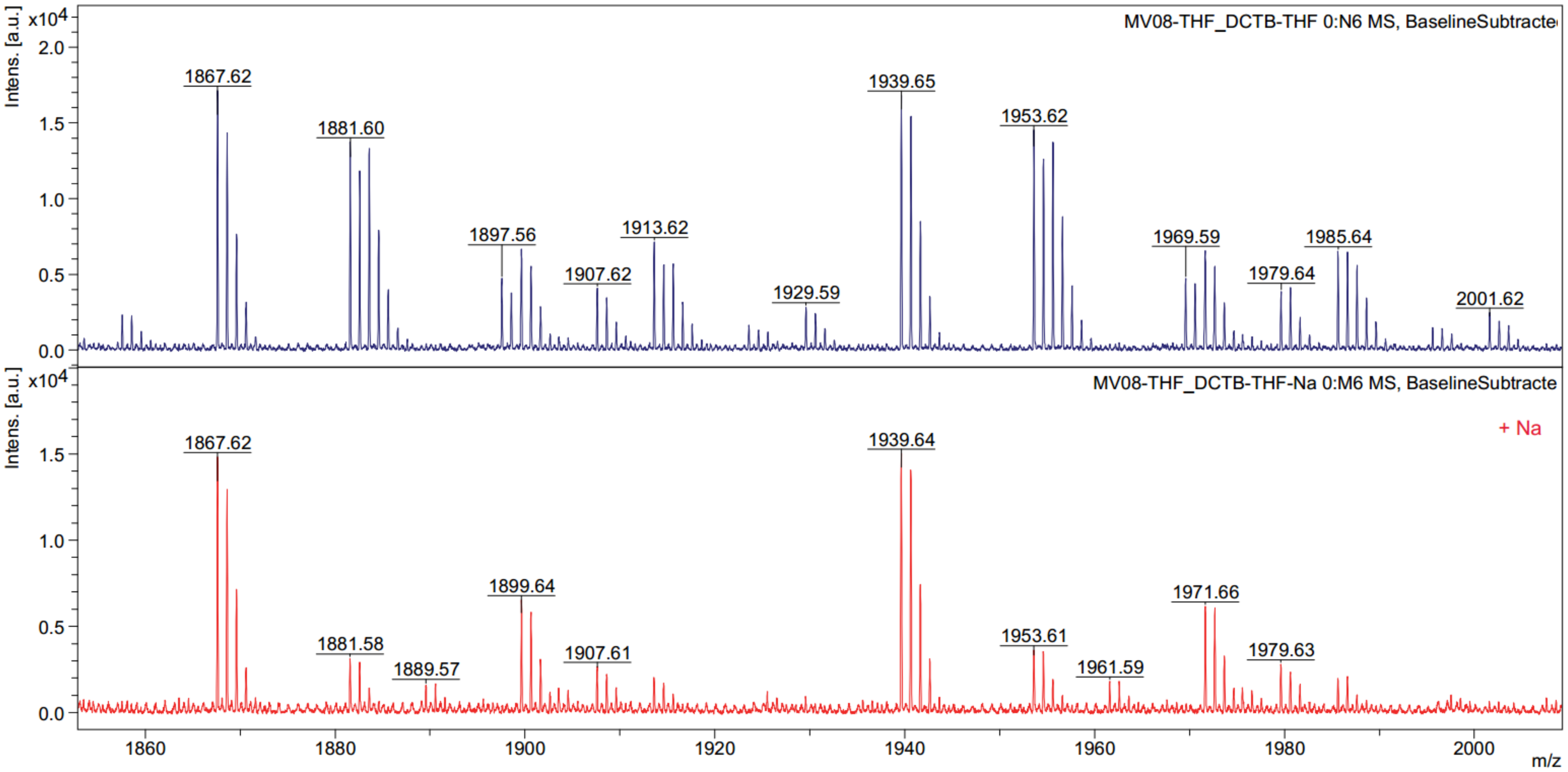

Figure S16. MALDI Mass spectrum of degraded PLA-ITA pellet at pH 5.3.

4. Degradation mechanism of PLA-ITA nanoparticles

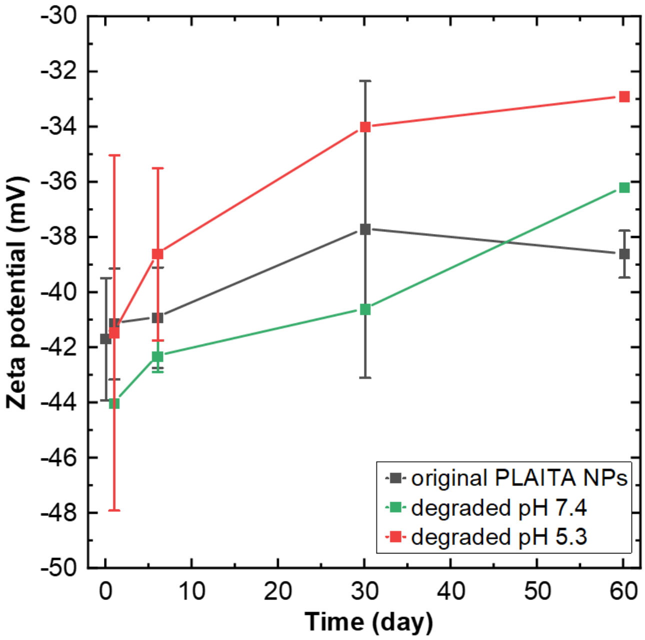

Figure S17**.** Evolution of zeta-potential of original and degraded PLA-ITA NPs suspension.

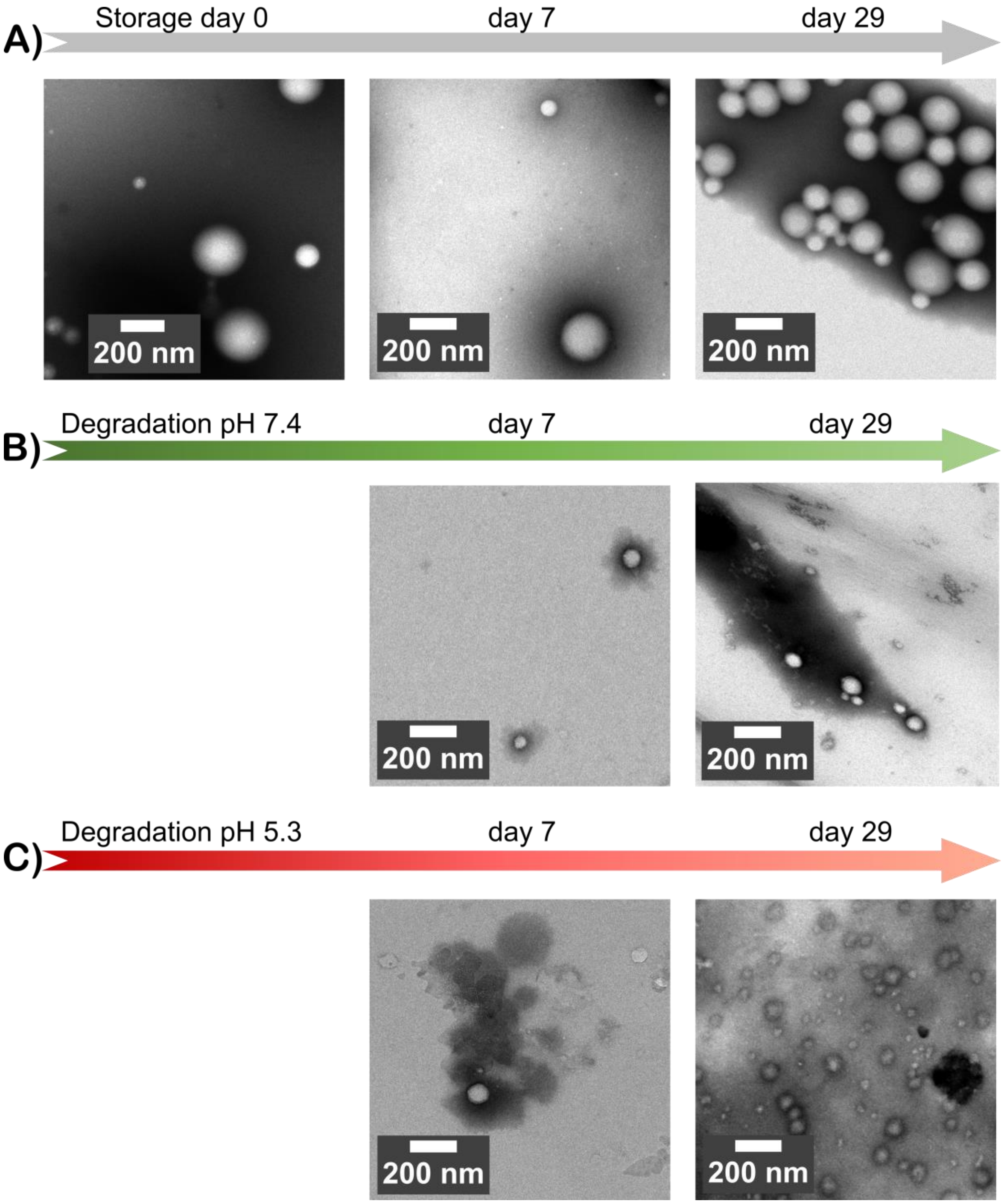

Figure S18. TEM images of PLA NPs in a 1-month course: (**A**) original, and degraded at (**B**) pH 7.4, and (**C**) pH 5.3.
